# Detected impacts of atmospheric rivers on marine heatwaves

**DOI:** 10.1038/s41467-026-74249-9

**Published:** 2026-06-11

**Authors:** Suqiong Hu, Shineng Hu

**Affiliations:** Division of Earth and Climate Sciences, Nicholas School of the Environment, Duke University, Durham, NC USA

**Keywords:** Atmospheric science, Climate change

## Abstract

Marine heatwaves (MHWs) are periods of unusually high sea surface temperature that can persist for weeks to months and extend across thousands of kilometers. Their increasing frequency and intensity under climate change threaten marine ecosystems and fisheries, yet the physical processes that govern their occurrence and evolution remain poorly understood. Here we analyze satellite and reanalysis data to show that atmospheric rivers (ARs)—long, narrow corridors of concentrated atmospheric moisture, often described as “rivers in the sky”—play a previously overlooked role in the development of MHWs in the North Pacific and North Atlantic. Under an AR, increased cloud cover cools the ocean through reduced solar radiation, while anomalously warm, humid air warms the ocean through the reduction of turbulent heat fluxes from the ocean. These two opposing mechanisms, dominant among others, vary with background climate state, causing seasonally and regionally varying ARs’ impacts on MHWs. These findings stress the importance of understanding ocean-atmosphere compound extremes and their changes under a warming climate.

## Abstract (web summary)

This study shows that atmospheric rivers (ARs) influence marine heatwaves in a seasonally dependent manner, driven by imbalances between AR-induced turbulent and radiative heat fluxes under varying seasonal background states.

## Introduction

The world’s oceans have absorbed over 90% of the excess heat due to anthropogenic greenhouse gas emissions, making ocean warming one of the most robust indicators of the ongoing climate change^1^. Against the backdrop of persistent ocean warming, marine heatwaves (MHWs)—discrete and prolonged periods of anomalously high ocean temperatures^2–4^, have emerged as a globally pervasive phenomenon with their frequency, duration, and intensity increasing rapidly^3,5–7^. Although MHW research has largely emphasized summer extremes, when absolute sea surface temperatures are high, MHWs are more fundamentally defined by deviations from the climatological mean and technically can occur in any season^4^. Many marine species acclimate to prevailing seasonal temperatures, which can reduce their tolerance to anomalous warming relative to the seasonal baseline^8–11^, and even moderate temperature anomalies may thus have important biological consequences. Therefore, MHWs can cause widespread ecological and economic disruptions year-round, including mass mortalities of marine organisms, severe impacts on fisheries, and major losses to coastal economies^12–14^. Yet despite growing impacts, the physical drivers governing the MHW development remain elusive^2,15,16^, limiting our ability to predict and mitigate their impacts.

Large-scale climate variability modes—such as the El Niño–Southern Oscillation (ENSO), the Pacific Decadal Oscillation (PDO), and the North Atlantic Oscillation (NAO)—have been suggested to modulate the likelihood and characteristics of MHWs by altering background oceanic and atmospheric states^17–22^. Besides the low-frequency climate modes, short-lived but intense weather systems, such as tropical cyclones and atmospheric blocking, have also been shown to potentially contribute to the development and persistence of MHWs^23–26^. As a key weather system in the mid-latitudes, atmospheric rivers (ARs)—often described as “rivers in the sky”—are characterized by long, narrow corridors of concentrated moisture transport^27–31^ and can cause extreme precipitation and flooding upon arrival at the continents, especially when impinging on mountains like in the western North America or Europe^18,32–34^. An AR spends most of its lifetime over the oceans, developing and migrating, and is often accompanied by strong surface winds and abundant tropospheric water vapor^34–36^. Given these atmospheric conditions, ARs have the potential to influence or exacerbate ocean temperature anomalies through radiative, thermodynamical, and dynamical effects^35,37–39^. It leads us to hypothesize that ARs can influence the development of MHWs on sub-monthly timescales. To the best of our knowledge, this MHW-AR connection has not been systematically studied before, despite its importance for ecosystems, fisheries and societies vulnerable to such events.

Aiming to fill this gap, in this study we use satellite observations and reanalysis datasets to investigate the impacts of ARs on MHWs and the underlying physical mechanisms. We focus particularly on the extratropical North Pacific and North Atlantic that stand out as hotspots where both MHWs and ARs are active and impactful^20,32,40,41^ (Supplementary Fig. 1), which directly points to the possibility of interactions between ARs and MHWs. As we show below, ARs have prominent, seasonally and regionally varying impacts on MHW development—an overlooked dimension of marine heatwave dynamics.

## Results

### The observed impacts of ARs on MHWs

**Fig. 1:**
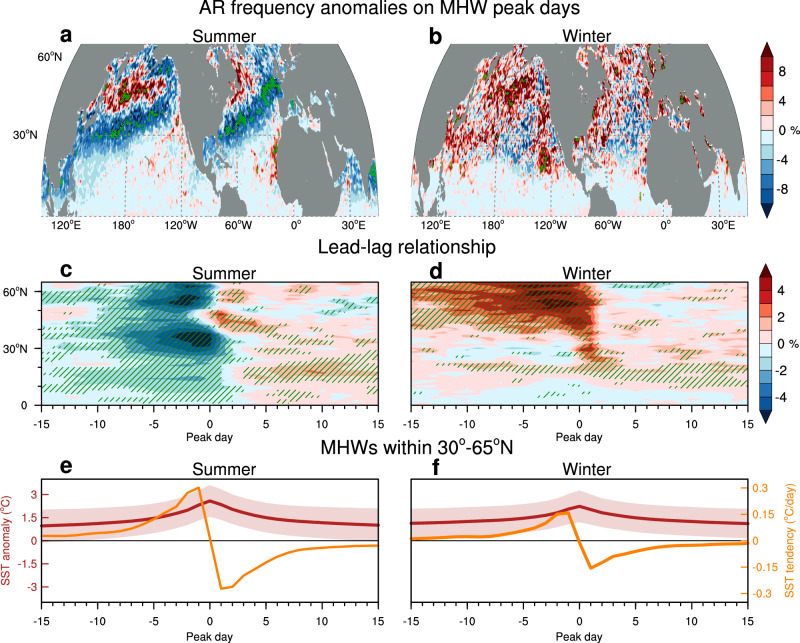
Relationship between atmospheric rivers (ARs) and marine heatwaves (MHWs). **a**,** b** Composite anomalies of daily AR frequency (%) on MHW peak days during boreal summer (June–August) and winter (December–February). **c**,** d** Composite anomalies of zonal-mean daily AR frequency (%) from 15 days before ( − 15) to 15 days after ( + 15) MHW peak days in summer and winter. Hatched regions indicate areas where composite anomalies are statistically significant at the 90% confidence level. **e**,** f** Composite of sea surface temperature (SST) anomalies (°C; red line) and the tendency of SST anomalies (°C/day; orange line) for all MHW events within the 30°N–65°N band, from 15 days before ( − 15) to 15 days after ( + 15) MHW peak days in boreal summer and winter. The pink shading indicates the spread ( ± 1 standard deviation) among the MHW events. The analysis period is 1982–2023 for all the panels.

All the MHWs and ARs in the North Pacific and the North Atlantic during 1982–2023 are identified with the criteria described in Methods. Then, the AR frequency anomalies on the MHW peak day are averaged across all the identified MHWs. This procedure is done separately for each ocean grid point and for boreal summer (June-August) and winter (December-February) so that seasonally varying spatial patterns of AR frequency anomalies can be revealed (Fig. 1a, b). The strongest signals of AR frequency anomalies are found in the extratropical oceans (30°N–65°N), where ARs are mostly active (Supplementary Fig. 1a). In summer, the MHW-associated AR frequency anomalies exhibit a horseshoe pattern over the North Pacific that has a positive center in the mid-latitude oceans surrounded by two negative lobes in the subtropical and subpolar oceans, respectively (Fig. 1a). Here, a negative AR frequency anomaly indicates that an anomalously low AR frequency is typically observed when a MHW peaks at the given location and vice versa. However, in winter, AR frequency anomalies show a completely different pattern with almost basinwide positive anomalies, which implies a consistently higher AR frequency associated with MHWs (Fig. 1b). Over the North Atlantic, similar large-scale structures are found for both seasons but overall weaker. Our analysis and discussion below will consider both basins.

To verify the robustness of the observed AR-MHW relationship, we further examine the composite AR frequency anomalies during MHW peak days using the historical simulations from 15 models participating in the Coupled Model Intercomparison Project Phase 6 (CMIP6) (Supplementary Table 1). The multi-model mean can reasonably reproduce the observed AR–MHW relationship, capturing both the summertime horseshoe-shaped pattern and the wintertime basin-wide structure (Supplementary Fig. 2). These features are robust across models, with more than 70% of them (11 out of 15 models) showing the same sign as the multi-model mean over most regions.

To explore a potential causal link, we further investigate the lead-lag relations between MHWs and ARs. We compute the composites of daily AR frequency anomalies during the 15 days before and after MHW peak days at each grid point and then compute the zonal average of the resulting composites (Fig. 1c, d). For both seasons, AR frequency anomalies tend to emerge more than a week in advance and reach the strongest magnitude about two days preceding the MHW peaks. MHW peaks are overall preceded by fewer ARs in summer (Fig. 1c), and more ARs in winter (Fig. 1d). These lead-lag relations are also found in CMIP6 historical simulations (Supplementary Fig. 2c, d). These results suggest that ARs influence the MHW development, probably through a cumulative effect prior to the peak of MHW events. We examined the AR frequency anomalies averaged during the 5 days preceding the MHW peak, and these results indeed show clearer and more spatially coherent spatial structures with an overall higher significance level than the AR frequency anomalies at the MHW peak, particularly over the North Pacific (Supplementary Fig. 3; also see Supplementary Fig. 4). The maximum SST tendency for the composite of all the MHWs in the midlatitude bands occurs about two days before the MHW peak (Fig. 1e, f), consistent with the lead time of AR frequency anomalies (Fig. 1c, d). In contrast, the post-MHW AR frequency anomalies are relatively weak.

**Fig. 2:**
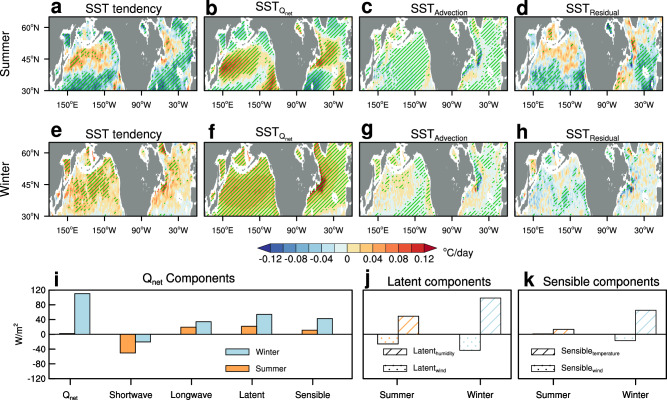
Spatial decomposition of sea surface temperature (SST) tendency based on ocean mixed-layer heat budget analysis and components of net surface heat flux (*Q*_*net*_) anomalies during atmospheric river (AR) days. **a**–**d** Composite SST tendency (°C/day) and the contributions from net surface heat flux (${{\rm{SST}}}_{{Q}_{{\rm{net}}}}$), horizontal oceanic advection (${{\rm{SST}}}_{{\rm{Advection}}}$), and the residual term (${{\rm{SST}}}_{{\rm{residual}}}$) during summer AR days over the period 1993–2023. **e**–**h** Same as (**a**–**d**), but for winter AR days. Hatched regions indicates areas where composite anomalies are statist**i**cally significant at the 90% confidence level. **i** Composites of ${Q}_{{\rm{net}}}$, net surface shortwave radiation, net surface longwave radiation, latent heat flux, and sensible heat flux anomalies (W/m^2^) during AR days in summer (orange bars) and winter (blue bars). **j** Decomposition of anomalous latent heat flux (W/m^2^) into contributions from wind speed anomalies (${{\rm{Latent}}}_{{\rm{wind}}}$) and air–sea specific humidity difference anomalies (${{\rm{Latent}}}_{{\rm{humidity}}}$). **k** Decomposition of anomalous sensible heat flux (W/m^2^) into contributions from wind speed anomalies (${{\rm{Sensible}}}_{{\rm{wind}}}$) and air–sea temperature difference anomalies (${{\rm{Sensible}}}_{{\rm{temperature}}}$).

The lead-lag relations above suggest that preceding ARs may have played an important role in the development of MHW events. To confirm that, we now change the reference frame to ARs and investigate the composite SST tendency on the days with high AR activity (Methods). The spatial structures of AR-induced SST tendency closely resemble those of AR frequency anomalies on MHW peaks, displaying a horseshoe-like cooling effect in summer and a basinwide warming effect in winter (Fig. 2a, e). Notably, the relatively weak wintertime AR-associated SST change over the North Atlantic is consistent with the weak MHW-associated AR frequency anomaly in this region (Fig. 1b and Fig. 2e). The results above support our hypothesis that ARs can influence the underlying MHW properties and further suggest that such influences can be spatially and seasonally dependent.

### Mechanisms of AR-induced SST changes

The pronounced seasonal contrast in AR-induced SST changes, which manifests as a horseshoe pattern in summer and a basinwide structure in winter, raises the question of what physical processes govern these distinct spatial structures. To answer this question, we conduct an ocean mixed-layer heat budget analysis on the AR days over the period of 1993–2023 with a daily, global ocean reanalysis dataset (Methods). Limited by data availability, this analysis period is shorter than that for our previous analysis of MHW-AR relation (i.e., 1982-2023), but the composite SST tendency patterns on AR days remain nearly unchanged between the two periods (Supplementary Fig. 5), confirming the robustness of these results.

Among the heat budget terms, net surface heat flux (${Q}_{{\rm{net}}}$) acts as the dominant driver of the AR-associated SST tendency in both summer and winter (Fig. 2b, f), resembling the observed SST tendency patterns (Fig. 2a, e) with a spatial correlation of 0.42 and 0.72 (*p* < 0.01), respectively. Horizontal oceanic advection has only a limited contribution in both seasons (Fig. 2c, g), and its large-scale feature is mainly shaped by the meridional Ekman advection associated with consistent surface wind changes (Supplementary Figs. 6 and 7). More specifically, a westerly (easterly) wind anomaly tends to induce an anomalous southward (northward) Ekman transport and therefore a cooling (warming) effect. Hsu et al.^37^. reported similar features of AR-associated oceanic heat advection in the North Pacific during October-March, and the small difference between our results comes mainly from the definition of seasons and the ocean reanalysis dataset used (Supplementary Fig. 8). The residual term mainly includes unresolved processes such as vertical entrainment, vertical mixing, horizontal diffusion, and other subgrid-scale variability. It may also partly arise from the fact that the surface heat flux and ocean fields during 1993–2018 are derived from different data sources (see “Methods” for a detailed discussion). The residual term shows a modest contribution (Fig. 2d, h) and exhibits generally similar spatial structures as the ${Q}_{{\rm{net}}}$ term but with an opposite, weaker magnitude, which implies a damping effect of oceanic processes as opposed to atmospheric forcing, broadly consistent with Hsu et al.^37^. To further quantify this oceanic damping effect, we computed basin-wide spatial correlations between the ${Q}_{{\rm{net}}}$-induced SST tendency and the SST tendency associated with the residual term. In winter, the correlations are –0.60 for the full study region, –0.58 for the North Atlantic, and –0.60 for the North Pacific. These strong negative correlations indicate that the residual term consistently offsets the basin-scale SST tendency pattern forced by ${Q}_{{\rm{net}}}$. In summer, the correlations are weaker (–0.34, –0.33, and –0.32, respectively), indicating a reduced but still evident damping effect. The residual term is also positively correlated with summertime mixed-layer depth (MLD) anomalies (r = 0.45), suggesting that regions with a deeper (shallower) ocean mixed layer tend to experience weaker (stronger) residual cooling. Such behavior is physically consistent with vertical mixing processes: when the mixed layer is shallow, the SST is more sensitive to mixing-induced cooling, leading to an enhanced cooling. Taken together, these results highlight the dominant role of net surface heat flux in regulating the SST response to ARs.

What are the detailed processes underlying the ARs’ influence on net surface heat flux? Why are these impacts dependent on season? To answer these questions, we decompose the net surface heat flux and quantify the contribution from each term of radiative and turbulent heat fluxes. Climatologically, net shortwave radiative flux is directed downward into the ocean, while net longwave radiative flux is upward (Supplementary Fig. 9b, c, g, h). The turbulent heat fluxes including latent and sensible heat fluxes are predominantly upward over most of the study region (Supplementary Fig. 9d, e, i, j), indicating a net heat transfer from the ocean to the atmosphere. An AR can influence MHW through all four components: it has a cooling effect through a reduced net downward shortwave radiative flux, and warming effects through a reduced net upward longwave radiative flux and weakened, or locally reversed, turbulent latent and sensible heat fluxes from the ocean to the atmosphere (Fig. 2i; also see Supplementary Fig. 10 for spatial structures). Specifically, the reduced net downward shortwave radiative flux is due mainly to the increase of clouds when ARs are active (Supplementary Fig. 11), while the reduced net upward longwave radiative flux is caused by the enhanced greenhouse effects of water vapor and clouds (Supplementary Fig. 12). The increase of surface wind speed associated with ARs tends to enhance ocean heat loss through turbulent latent and sensible heat fluxes, but this cooling effect is outweighed by the warming effect of the warm, moist air transported by ARs through the enhanced downward turbulent heat fluxes (Fig. 2j, k; also see Supplementary Fig. 13 for spatial structures). These characteristics are generally consistent with the annual-mean, AR-related surface heat flux anomalies shown by ref. ^38^., and below we further discuss the distinct features for winter and summer, separately.

**Fig. 3:**
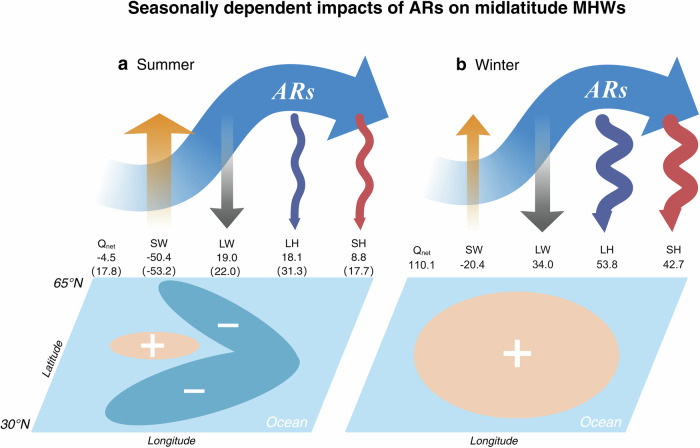
Mechanistic schematic for the fingerprints of atmospheric rivers (ARs) on marine heatwaves (MHWs). Seasonally varying fingerprints of ARs on the development of MHWs are detected in the North Pacific and Atlantic (30°–65°N). The impacts of ARs on the underlying ocean temperature exhibit a horseshoe-shaped pattern in boreal summer (**a**) and a basinwide pattern (**b**) in boreal winter. The decomposition of AR-induced net surface heat flux (${Q}_{{\rm{net}}}$) anomalies averaged over 30°–65°N is also shown. SW, LW, LH, and SH denote net surface shortwave radiation, net surface longwave radiation, latent heat flux and sensible heat flux, respectively. See text for details. See text for details of the individual heat flux components (W/m^2^). In panel (**a**), values inside and outside the parentheses correspond to the negative and positive sea surface temperature (SST) tendency regions, respectively.

The seasonal contrast in AR-associated net surface heat flux change results from a delicate compensation among these competing cooling and warming effects that can each independently vary with season. The cloud-induced shortwave radiative cooling effect associated with ARs is particularly strong in summer when the background incoming solar radiation is large (Supplementary Fig. 11). The greenhouse effects of water vapor and clouds associated with ARs are stronger in winter as the background atmospheric emissivity is relatively small and so there is less overlap of longwave absorption band (Supplementary Fig. 12). The effects of AR-associated warm and moist air on surface turbulent heat fluxes are stronger in winter (Fig. 2j, k; and Supplementary Fig. 13), caused by both stronger background winds and enhanced air–sea humidity and temperature contrasts (Supplementary Figs. 14 and 15). In summary, modulated by the background atmospheric state, the AR-associated warming effects through turbulent and longwave radiative fluxes are stronger in winter, while the AR-associated shortwave cooling effect is stronger in summer. The collective effect of ARs in the North Pacific and the North Atlantic is to generally suppress MHWs in summer, except for the mid-latitude band (~40-50°N), but to favor MHWs in winter (Fig. 3).

To further elucidate the horseshoe-shaped summer pattern, we decomposed the ${Q}_{{\rm{net}}}$ anomalies over the positive and negative SST tendency regions shown in Fig. 2a. As shown in Supplementary Fig. 16, during summer, the shortwave and longwave radiative flux anomalies associated with AR events are comparable between the two areas. In contrast, turbulent heat flux anomalies (including both sensible and latent components) are much stronger in the midlatitude positive SST tendency band, ~twice those in the negative SST tendency region, which is in turn due mainly to the enhanced air-sea temperature and humidity contrast anomalies (Supplementary Fig. 15a, c). The enhanced warming effect associated with the turbulent heat flux anomalies acts to offset the cooling effect associated with the shortwave radiative flux anomalies, resulting in a net surface heat gain in the midlatitude band and therefore a horseshoe-shaped pattern in summer.

## Discussion

In this study, we have identified ARs’ seasonally dependent fingerprints on the MHWs in the North Pacific and Atlantic through observational analysis, as summarized in Fig. 3. When ARs are present, the associated clouds tend to cool the ocean by reducing the downward shortwave radiative flux, while the warm and moist air tends to warm the ocean through both reducing turbulent heat fluxes (both latent and sensible) from the ocean to the atmosphere and increasing downward longwave radiative flux. Modulated by the background atmospheric state, these flux components delicately work together to yield a seasonally dependent net effect. AR frequency anomalies precede MHW peaks by several days with distinct spatial signatures—a horseshoe-shaped pattern with mainly negative anomalies in summer and a basinwide pattern with mainly positive anomalies in winter. In addition to surface heat fluxes, AR-associated precipitation may potentially influence upper-ocean thermal variability through freshwater-induced stratification and changes in vertical mixing^42,43^. However, isolating the independent contribution of AR-related precipitation on SST variability from observational datasets is challenging. Quantifying this effect will require targeted climate model simulations that warrant future investigations.

While background ocean warming sets the stage for more frequent and intense MHWs, ARs can modulate their timing, intensity, and spatial structure on synoptic scales. Recognizing ARs as a key forcing mechanism for MHWs advances our understanding of extreme ocean temperature variability and underscores the need for further investigation into their role in MHW dynamics. Beyond this one-way influence, previous studies have suggested the possibility of feedbacks from MHWs to AR characteristics. For example, a recent study focusing on MHWs in the northeastern Pacific showed that ARs following MHW peak days tend to be stronger and shifted northeastward compared to ARs not associated with MHWs^44^. In addition, case-based studies indicated that anomalously warm SST may enhance moisture convergence along AR pathways^45^. Together, these results point to potential two-way interactions between ARs and MHWs; however, quantifying such feedbacks is beyond the scope of the present study and will require targeted investigation using fully coupled atmosphere–ocean models.

ARs are closely linked to synoptic atmospheric variability in the extra-tropics, including extratropical cyclones, storm tracks, atmospheric blocking systems, jet streams, etc^46–48^. Our findings highlight AR variability as an important but previously overlooked atmospheric pathway that connects MHW events to these large-scale atmospheric circulation variability in the extratropical oceans. For example, atmospheric blocking, defined as a quasi-stationary high-pressure system, exerts a strong influence on MHWs through reduced wind speed and increased shortwave radiation^40,49^. At the same time, these persistent anticyclonic anomalies can alter large-scale flow and moisture distribution, therefore modulating the propagation and intensity of ARs^48,50,51^. Whether ARs can feed back onto the synoptic circulation variability through moisture convergence and the interactions with MHWs is not fully understood.

Our results further suggest that the changes in AR characteristics under a warming climate may influence the occurrence and intensity of MHWs in the extra-tropics^52–54^. Conversely, intensifying MHWs under global warming may in turn feed back onto the atmospheric circulation to reshape AR activity^45,55,56^. The interaction between ARs and MHWs may represent a key pathway for the emergence of compound ocean–atmosphere extremes under future warming, critical for marine ecosystems and regional hydroclimate. Future research is needed to assess the two-way mutual interactions between MHWs and ARs in observations and coupled climate models, and to explore the potential to improve the short-term forecast and long-term projection of MHWs by accounting for these interactions.

## Methods

### Datasets

MHWs are analyzed using the NOAA Daily Optimum Interpolation Sea Surface Temperature dataset, version 2.1 (DOISST v2.1)^57^. The original daily SST data, available at 0.25° × 0.25° resolution, are regridded to a regular 1° × 1° grid by spatial averaging within each 1° cell prior to the calculation of MHW characteristics. Daily atmospheric fields are obtained from the fifth major global reanalysis produced by European Center for Medium-Range Weather Forecasts (ECMWF) v5 (ERA5) dataset, with a horizontal resolution of 1° × 1°^58^. Variables used include net surface sensible heat flux, latent heat flux, shortwave and longwave radiative fluxes (both all-sky and clear-sky), 2-m air temperature, specific humidity, and 10-m horizontal winds. The analysis spans the period of 1982–2023, with anomalies computed relative to the climatological mean over the same period. Daily SST outputs from the CMIP6 historical simulations are used to identify MHW peak days in 15 selected models (Supplementary Information Table 1). Daily specific humidity and pressure-level horizontal winds from each model are used to calculate vertically integrated water vapor transport (IVT). To ensure consistency among models, only one ensemble member (r1i1p1f1) from each model is included in the analysis. The model analyses cover the period 1980–2014.

To investigate the mechanisms underlying the seasonal contrast in AR-associated SST responses, we perform an ocean mixed layer heat budget analysis using the GLORYS12V1 global ocean reanalysis^59^ together with surface heat fluxes from ERA5. GLORYS12V1 is based on the operational global forecasting system of the Copernicus Marine Environment Monitoring Service (CMEMS) and employs the NEMO ocean model, which is forced at the surface by atmospheric fluxes from ECMWF reanalysis—ERA-Interim before 2018 and ERA5 after 2019. We recalculated the ocean mixed layer heat budget in Fig. 2 but for the period of 2019–2023, during which there was no inconsistency issue (i.e., GLORYS12V1 is forced by ERA5). Overall, the results (Supplementary Fig. 17) are similar to those for the entire period (Fig. 2), but much noisier due to the shorter period and therefore the smaller sample size being used. Also, we examined the ${Q}_{{\rm{net}}}$-induced SST tendency over the entire period, as well as separately before 2018 and after 2019. The spatial patterns and seasonal characteristics are overall very similar for those three periods (Supplementary Fig. 18). These tests provide us additional confidence in the robustness of our results, although they do not completely eliminate the inherent uncertainty in interpreting the residual term. Therefore, ERA5 is used consistently in this study to derive AR-related surface heat fluxes and atmospheric variables across the full analysis period, ensuring a uniform atmospheric reference framework.

GLORYS12V1 provides daily output at 1/12° horizontal resolution with 50 vertical levels and spans the satellite altimetry era beginning in 1993. The variables used in this study include mixed-layer depth (MLD), ocean potential temperature, and ocean currents. In GLORYS12V1, the MLD is diagnosed using a density-based criterion and is defined as the depth at which the potential density increase relative to the density at 10 m corresponds to a temperature decrease of 0.2 °C under local surface conditions.

### Statistical analysis

To evaluate the robustness of the AR–MHW relationship identified in this study, statistical significance tests were applied to the composite fields using a two-tailed Student’s t-test. In addition, to examine whether the identified relationship is influenced by long-term trends, all analyses were repeated using linearly detrended datasets at each grid point. The main conclusion remains unchanged, indicating that the results are not driven by long-term variability (Supplementary Fig. 19).

### MHWs definition

A MHW is defined as a discrete, prolonged, and anomalously warm ocean event, occurring when daily SST exceeds a seasonally varying 90th percentile threshold for at least five consecutive days^4^. The threshold is computed for each calendar day by first estimating the 90th percentile of daily SST using data within an 11-day window centered on that day across all years. To ensure temporal continuity, the resulting daily threshold is subsequently smoothed using a 31-day moving average. Events separated by fewer than three days are treated as a single MHW. The peak day of each event is defined as the day on which SST reaches its maximum during the event. For DOISST-based analysis, the climatological period is 1982–2023, whereas for GLORYS-based analysis, the threshold is computed over the 1993–2023 period. Based on these peak days, composites of AR frequency anomalies are computed by averaging daily AR frequency anomalies across all identified MHW peak days in each grid. Composites are constructed separately for boreal summer and winter, enabling assessment of seasonal contrasts in AR activity associated with extreme ocean warming (Fig. 1).

### ARs definition

Multiple AR detection algorithms have been proposed before (see a review in ref. ^60^). In this study, we use the ARs identified using a detection algorithm based on IVT, calculated from ERA5 6-h wind and specific humidity fields, vertically integrated from 1000 to 300 hPa on a 0.25° × 0.25° global grid^61^. A grid cell is tagged as part of an AR if the IVT exceeds the location- and month-specific 85th percentile threshold that is computed with a five-month moving window centered on each calendar month. To qualify as an AR, a group of tagged grid cells must have a total length exceeding 2000 km and a length-to-width ratio of at least 2. For consistency with the analysis framework, the AR detection results are regridded to a 1° × 1° spatial resolution. Given the use of 6-h input data, up to four AR detections may occur at each grid cell per day. Daily AR frequency is defined as the fraction of these 6-h time steps during which AR conditions are detected at each grid cell. A calendar day is classified as an “AR day” at a given grid point if AR conditions are detected in more than two of the four 6-h time steps within that day, regardless of whether the detections are consecutive. To assess the oceanic response to AR activity, we compute composites of SST tendency—defined as the day-to-day change in SST—on AR days. These composites are constructed separately for boreal summer and winter.

To better assess the robustness of the AR–MHW relationship to AR detection algorithms, we additionally analyzed the ARs detected by two independent AR detection algorithms: AR-CONNECT^62^, which is an object-based tracking algorithm using absolute IVT thresholds, and Lora v2^63,64^, a hybrid relative–absolute detection method that accounts for background climate variability. The spatial structures and seasonal contrasts are overall qualitatively similar among these algorithms, although quantitative differences in AR anomaly magnitude are present (Supplementary Fig. 20 and Supplementary Fig. 21).

### Mixed-layer heat budget

To understand atmospheric and oceanic processes responsible for SST variability during AR and MHW events, we conduct an ocean mixed-layer heat budget analysis. The ocean surface temperature tendency is expressed as:

1 $$\frac{\partial T}{\partial t}=\frac{{Q}_{{\rm{net}}}}{\rho {C}_{p}h}-{\bf{V}}\cdot \nabla T+\varepsilon$$

where $T$ is the SST, $\rho$ is the seawater density, ${C}_{p}$ is the specific heat capacity of seawater, and ℎ is the ocean mixed-layer depth. ${Q}_{{\rm{net}}}$ denotes the net surface heat fluxes, and $-{\bf{V}}\cdot \nabla {T}$ represents the horizontal advection of temperature within the mixed layer. All mixed-layer heat budget terms are computed diagnostically in this study. Specifically, daily mixed-layer depth, ocean potential temperature, and ocean currents are obtained from the GLORYS12V1 reanalysis, while the net surface heat flux is derived from the ERA5 reanalysis. The horizontal advection term is calculated using daily horizontal currents and temperature gradients within the mixed layer. The residual term $\varepsilon$ is estimated as the imbalance of the heat budget and represents the combined effects of unresolved processes within the mixed layer, including vertical entrainment, vertical mixing, horizontal diffusion, and other subgrid-scale processes.

### Linear decomposition of sensible and latent heat flux anomalies

Latent and sensible turbulent heat flux anomalies can be linearly decomposed using the bulk aerodynamic formulations. Specifically, the latent heat flux (LH) and sensible heat flux (SH) anomalies are approximated as:

2 $${{LH}}^{{\prime} }=\rho {L}_{v}{C}_{e}{\left[U\left({{q}_{s}-q}_{a}\right)\right]}^{{\prime} }\approx \rho {L}_{v}{C}_{e}{U}^{{\prime} }\left({{\bar{q}}_{s}-\bar{q}}_{a}\right)+\rho {L}_{v}{C}_{e}\bar{U}\left({q}_{s}^{{\prime} }-{q}_{a}^{{\prime} }\right)$$

3 $${{SH}}^{{\prime} }=\rho {c}_{p}{C}_{h}{\left[U({T}_{s}{-T}_{a})\right]}^{{\prime} }\approx \rho {c}_{p}{C}_{h}{U}^{{\prime} }({{\bar{T}}_{s}-\bar{T}}_{a})+\,\rho {c}_{p}{C}_{h}\bar{U}({T}_{s}^{{\prime} }-{T}_{a}^{{\prime} })$$

where $\rho$ is air density, ${L}_{v}$ is the latent heat of vaporization, ${C}_{p}$ is the specific heat capacity of air at constant pressure, and ${C}_{e}$, ${C}_{h}$ are the bulk transfer coefficients for moisture and heat, respectively. $U$ denotes wind speed, ${q}_{s}$ and ${q}_{a}$ are the specific humidity at the surface and in the overlying air, and ${T}_{s}$ and ${T}_{a}$ are the sea surace and near-surface air temperatures. Prime $({\prime})$ indicates anomalies, and overbar denotes climatological means. This decomposition separates the contributions from wind anomalies and air-sea specific humidity (or temperature) anomalies.

## Supplementary information

Supplementary information

Transparent Peer Review file

## Data Availability

All raw data used in this study are publicly available. Daily sea surface temperature (SST) data were obtained from the NOAA OISST v2.1 dataset (https://psl.noaa.gov/data/gridded/data.noaa.oisst.v2.highres.html). Atmospheric river (AR) data were obtained from the UCLA AR database (https://dataverse.ucla.edu/dataverse/ar). ERA5 atmospheric reanalysis data were retrieved from the Copernicus Climate Data Store (https://cds.climate.copernicus.eu/datasets). GLORYS ocean reanalysis data were obtained from the Copernicus Marine Service (https://data.marine.copernicus.eu/product/GLOBAL_MULTIYEAR_PHY_001_030/services). CMIP6 model output is available through a distributed data archive developed and operated by the Earth System Grid Federation (https://esgf-node.llnl.gov/projects/cmip6/). Processed data used for the analysis and main figure generation in this study is publicly available at (10.6084/m9.figshare.32336352).

## Code availability

Code for generating the main figures in this study is publicly available at (10.6084/m9.figshare.32336352).
