# Supplementary material for: Detected impacts of atmospheric rivers on marine heatwaves
Source: Nat Commun. 2026 Jun 11;17:7450. doi: 10.1038/s41467-026-74249-9 (PMC13408837; doi:10.1038/s41467-026-74249-9)
Supplement: Supplementary file 1 — Supplementary information [file 41467_2026_74249_MOESM1_ESM.pdf]

Supplementary Information

Detected Impacts of Atmospheric Rivers on Marine Heatwaves

Suqiong Hu[1], Shineng Hu[1*]

[1]Division of Earth and Climate Sciences, Nicholas School of the Environment, Duke University, Durham, NC, USA

[*]Corresponding author. Email: shineng.hu@duke.edu

This PDF file includes:
Supplementary Tables 1
Supplementary Figs. 1–21

Supplementary Table 1. Utilized historical simulations from 15 CMIP6 models

Models	Institution, Country
ACCESS-ESM1-5	CSIRO-ARCCSS, Australia
ACCESS-CM2	CSIRO-ARCCSS, Australia
CanESM5	CCCma, Canada
CESM2-FV2	NCAR, USA
CESM2	NCAR, USA
CESM2-WACCM	NCAR, USA
MIROC6	INM, Russia
MPI-ESM-1-2-HAM	MPI-M, Germany
MPI-ESM1-2-HR	MPI-M, Germany
MPI-ESM1-2-LR	MPI-M, Germany
MRI-ESM2-0	MRI, Japan
NESM3	NUIST, China
NorESM2-LM	NCC, Norway
NorESM2-MM	NCC, Norway
SAM0-UNICON	SNU, Republic of Korea

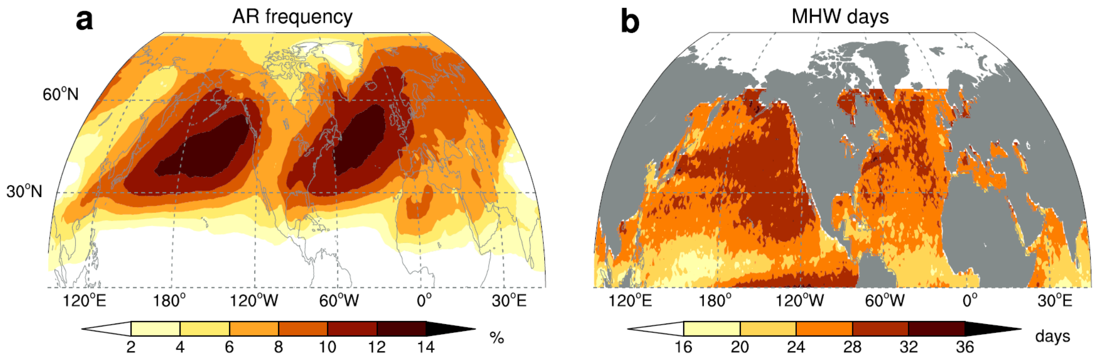

Supplementary Fig. 1. Spatial distribution of atmospheric river (AR) frequency and marine heatwave (MHW) days. Climatological spatial distribution of **(a)** annual mean AR frequency (%) and **(b)** MHW days (day), for the period 1982–2023. MHW days are not calculated for regions north of 65°N because of uncertainties associated with sea-ice coverage.

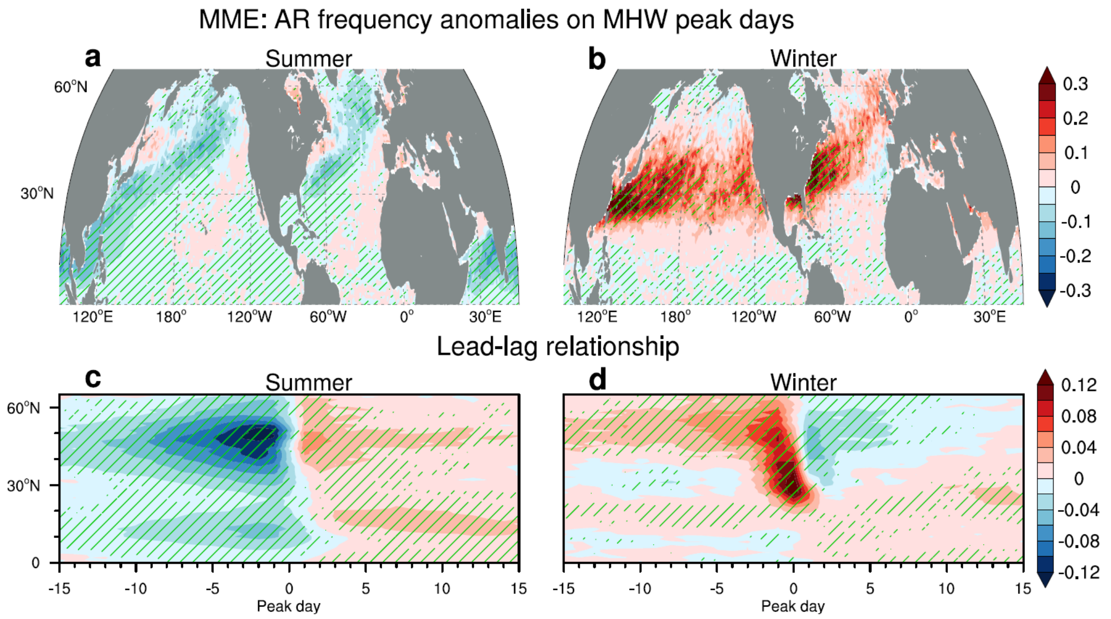

Supplementary Fig. 2. Relationship between atmospheric rivers (ARs) and marine heatwaves (MHWs) in historical simulations from 15 CMIP6 models. (a, b) Composite anomalies of daily AR frequency (%) on MHW peak days during boreal summer (June–August) and winter (December–February). **(c, d)** Composite anomalies of zonal-mean daily AR frequency (%) from 15 days before (−15) to 15 days after (+15) MHW peak days in summer and winter. Hatched regions indicate areas where more than 70% of models (≥11 out of 15 models) exhibit the same sign as the multi-model ensemble mean (MME).

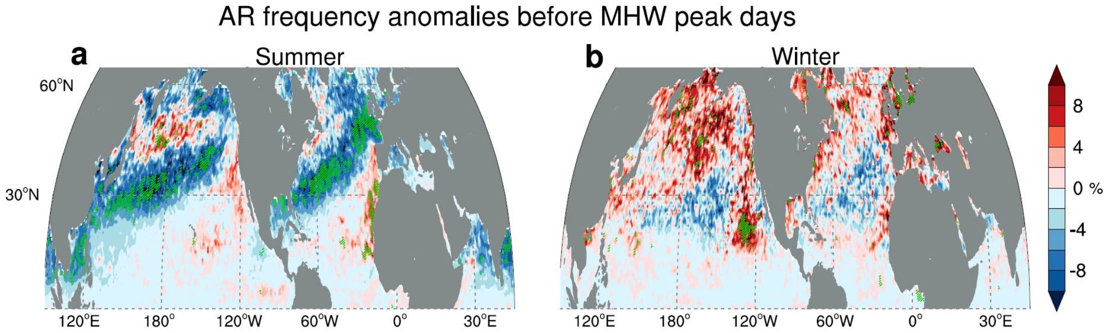

Supplementary Fig. 3. Atmospheric river (AR) frequency anomalies preceding marine heatwave (MHW) peak days. Composite AR frequency anomalies averaged over the five days before MHW peaks in **(a)** summer and **(b)** winter. Hatched regions indicate areas where anomalies are statistically significant at the 90% confidence level.

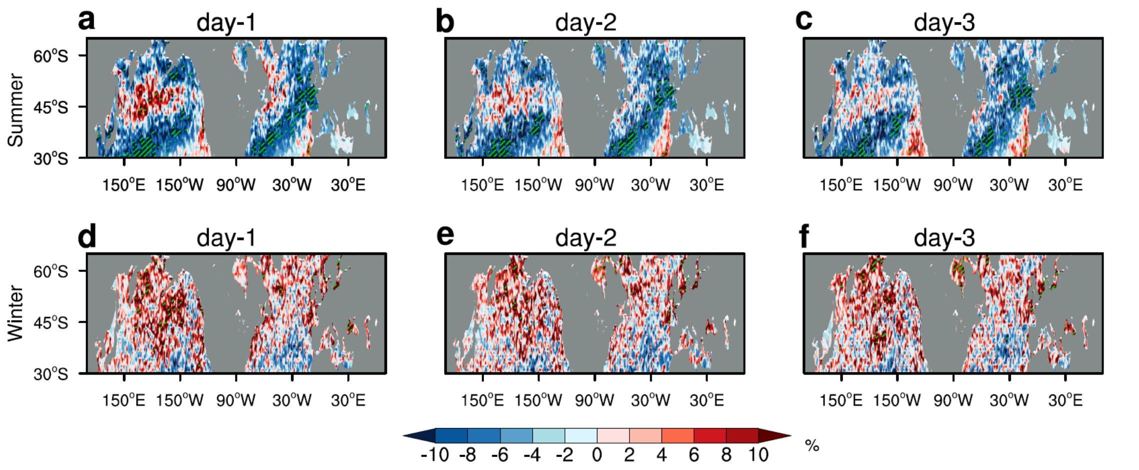

Supplementary Fig. 4. Atmospheric river (AR) frequency anomalies preceding marine heatwave (MHW) peak days. (a–c) Composites of daily AR frequency anomalies (%) since 3 days prior (day –3) to the peak of MHW events during boreal summer for the period of 1982–2023. **(d-f)** Same as **(a-c)**, but during winter. Hatched regions indicates areas where composite anomalies are statistically significant at the 90% confidence level.

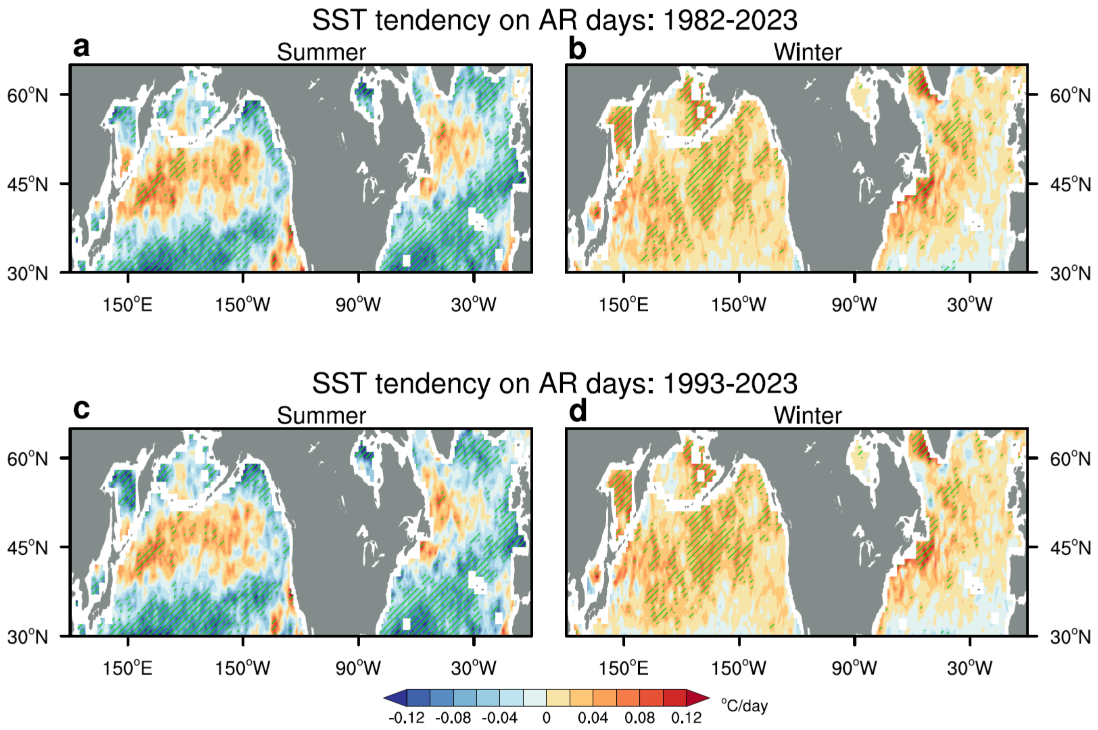

Supplementary Fig. 5. Sea surface temperature (SST) tendency during atmospheric river (AR) days. (a, b) Composite of SST tendency (°C/day) on AR days in boreal summer and winter for the period of 1982-2023. **(c, d)** Same as **(a, b)** but for the period of 1993-2023 as a sensitivity test. Hatched regions indicates areas where composite anomalies are statistically significant at the 90% confidence level.

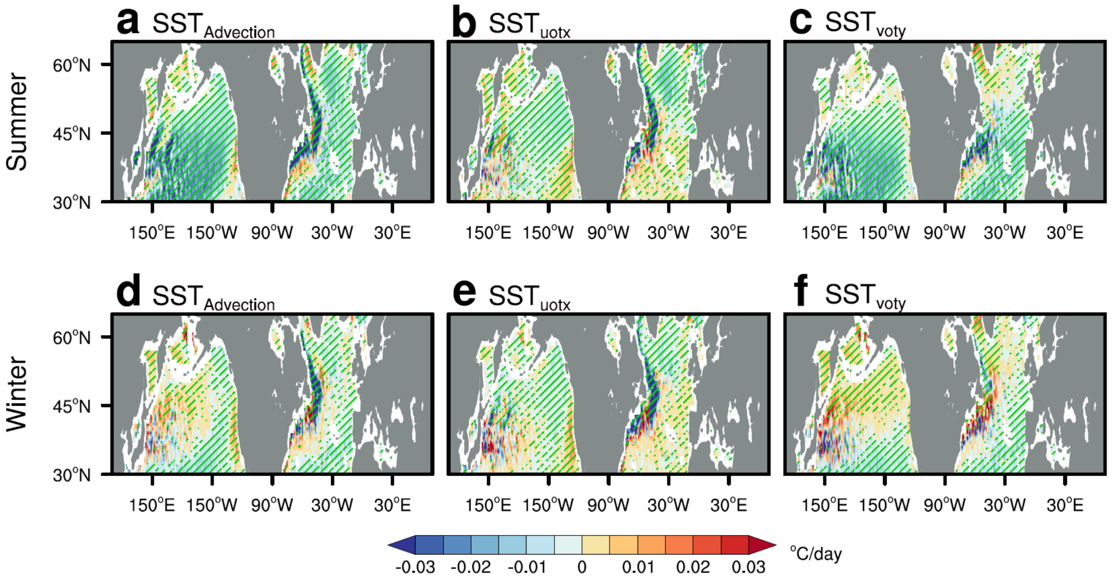

Supplementary Fig. 6. Horizontal oceanic advection ($SST_{Advection}$) anomalies during atmospheric river (AR) days. Composite anomalies (°C/day) of **(a)** horizontal oceanic advection, **(b)** its zonal component (SST_{uotx}), and **(c)** its meridional component (SST_{voty}) on summer AR days.**(d-f)** Same as **(a-c)**, but for winter AR days. Hatched regions indicate areas where composite anomalies are statistically significant at the 90% confidence level.

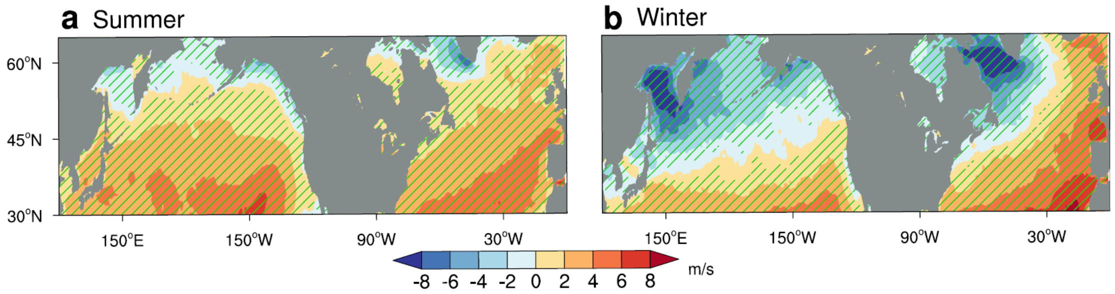

Supplementary Fig. 7. Atmospheric river (AR)-associated 10 m zonal wind anomalies. Composite 10 m zonal wind anomalies (shading; m/s) on **(a)** summer and **(b)** winter AR days. Hatched regions indicate areas where composite anomalies are statistically significant at the 90% confidence level.

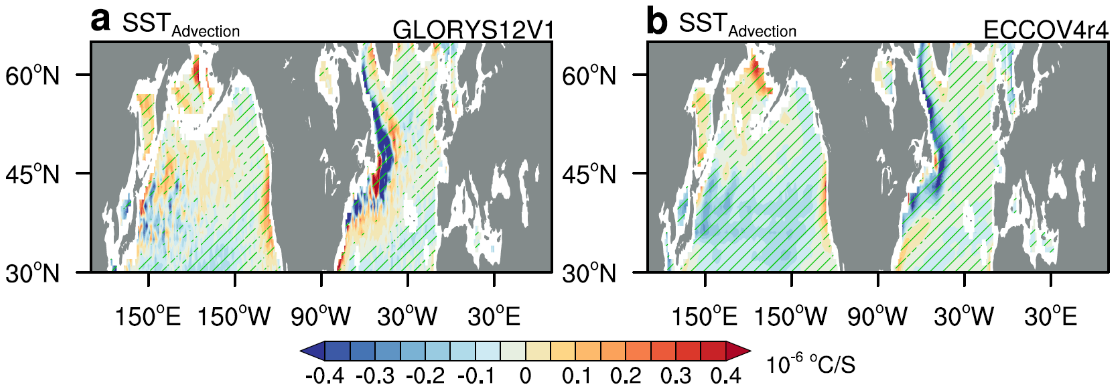

Supplementary Fig. 8. Horizontal oceanic advection ($SST_{Advection}$) anomalies during winter atmospheric river (AR) days. Composite anomalies (10^{-6} °C/S) of horizontal oceanic advection on winter (October–March) AR days using **(a)** GlORYS12V1 and **(b)** ECCOV4r4. Hatched regions indicate areas where composite anomalies are statistically significant at the 90% confidence level.

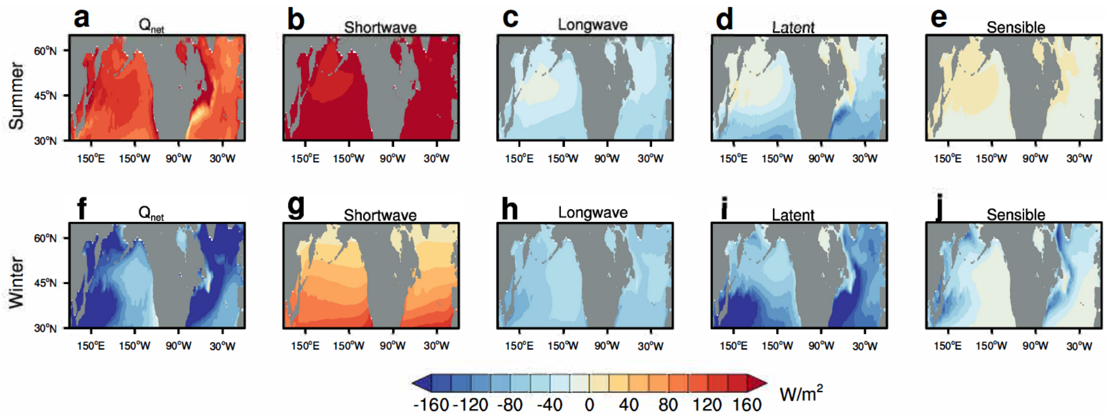

Supplementary Fig. 9. Decomposition of net surface heat flux (Q_{net}) climatology. (a–e) Climatology (W/m²) of Q_{net}, net surface shortwave radiation, net surface longwave radiation, latent heat flux, and sensible heat flux during summer. **(f–i)** Same as **(a-e)**, but for winter.

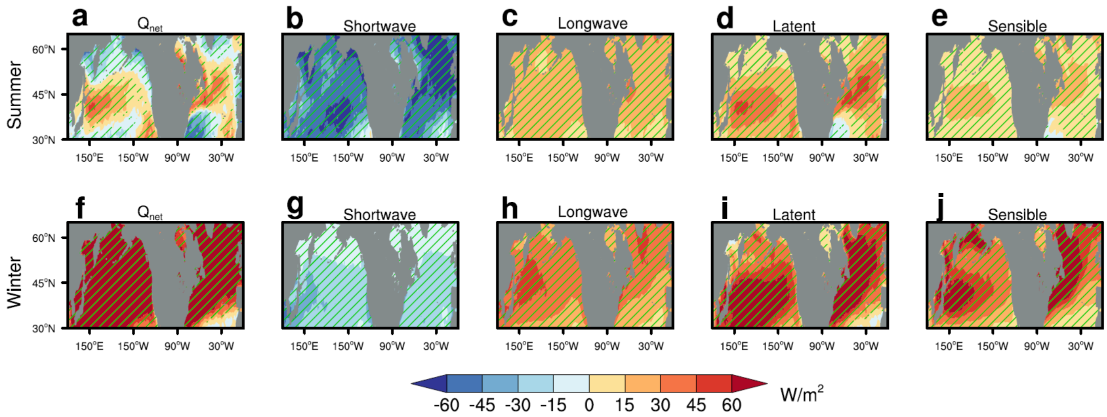

Supplementary Fig. 10. Decomposition of net surface heat flux (Q_{net}) anomalies on atmospheric river (AR) days. (a–e) Composite anomalies (W/m²) of Q_{net}, shortwave radiation, net longwave radiation, latent heat flux, and sensible heat flux during summer AR days. **(f–i)** Same as **(a-e)**, but for winter AR days. Hatched regions indicate areas where composite anomalies are statistically significant at the 90% confidence level.

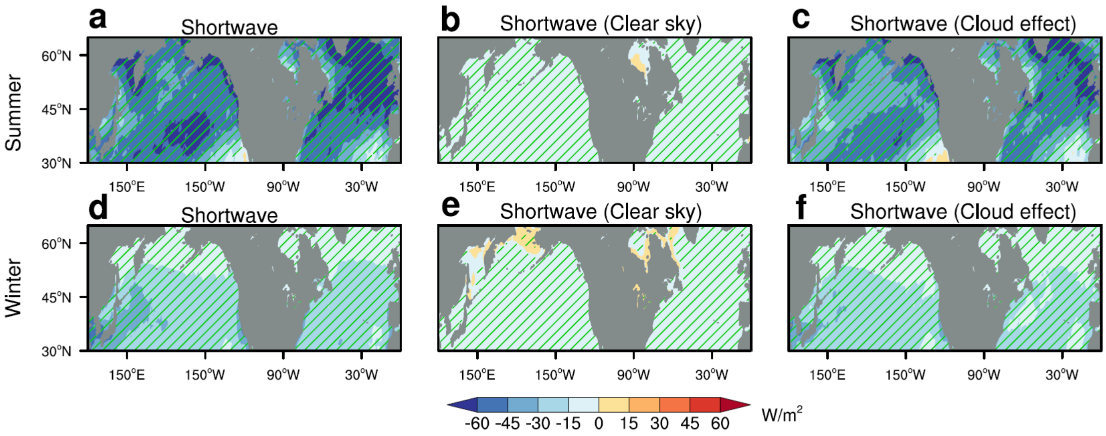

Supplementary Fig. 11. Shortwave radiation anomalies associated with atmospheric river (AR) days. (a) Composite anomalies (W/m²) of total shortwave radiation during summer AR days. **(b)** and **(c)** show the contributions from clear-sky and cloud-related components, respectively. **(d–f)** Same as **(a–c)**, but for winter AR days. Hatched regions indicate areas where composite anomalies are statistically significant at the 90% confidence level.

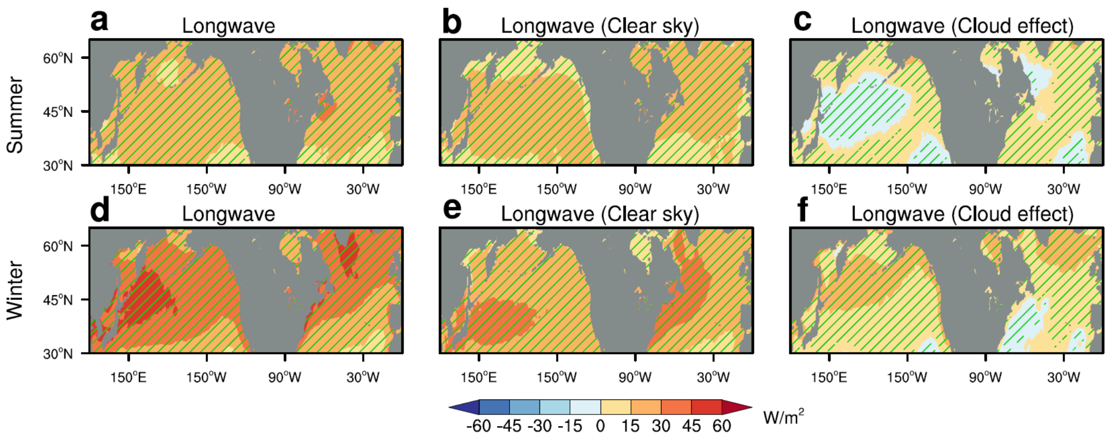

Supplementary Fig. 12. Longwave radiation anomalies associated with atmospheric river (AR) days. (a) Composite anomalies (W/m²) of total longwave radiation during summer AR days. **(b)** and **(c)** show the contributions from clear-sky and cloud-related components, respectively. **(d–f)** Same as **(a–c)**, but for winter AR days. Hatched regions indicate areas where composite anomalies are statistically significant at the 90% confidence level.

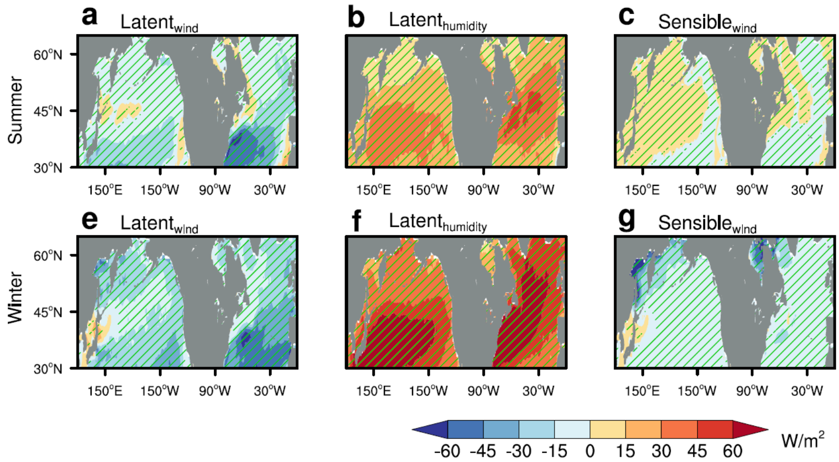

Supplementary Fig. 13. Linear decomposition of turbulent heat flux anomalies associated with atmospheric river (AR) days. (a, b) Composite anomalies (W/m²) of net surface latent heat flux components attributable to wind speed anomalies ($\mathrm{Latent_{wind}}$) and humidity gradient anomalies ($\mathrm{Latent_{humidity}}$) during summer AR days. **(c, d)** Composite anomalies (W/m²) of net surface sensible heat flux components attributable to wind speed anomalies ($\mathrm{Sensible_{wind}}$) and temperature gradient anomalies ($\mathrm{Sensible_{temperature}}$) during summer AR days. **(e, f)** Same as **(a, b)**, but for winter AR days. **(g, h)** Same as **(c, d)**, but for winter AR days. Hatched regions indicate areas where composite anomalies are statistically significant at the 90% confidence level.

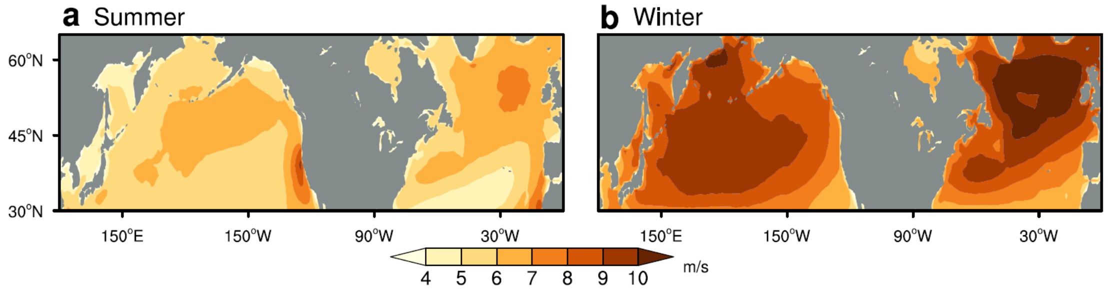

Supplementary Fig. 14. Climatology of 10 m wind speed. (a, b) Climatology of 10 m wind speed (m/s) during boreal summer and winter during 1982-2023.

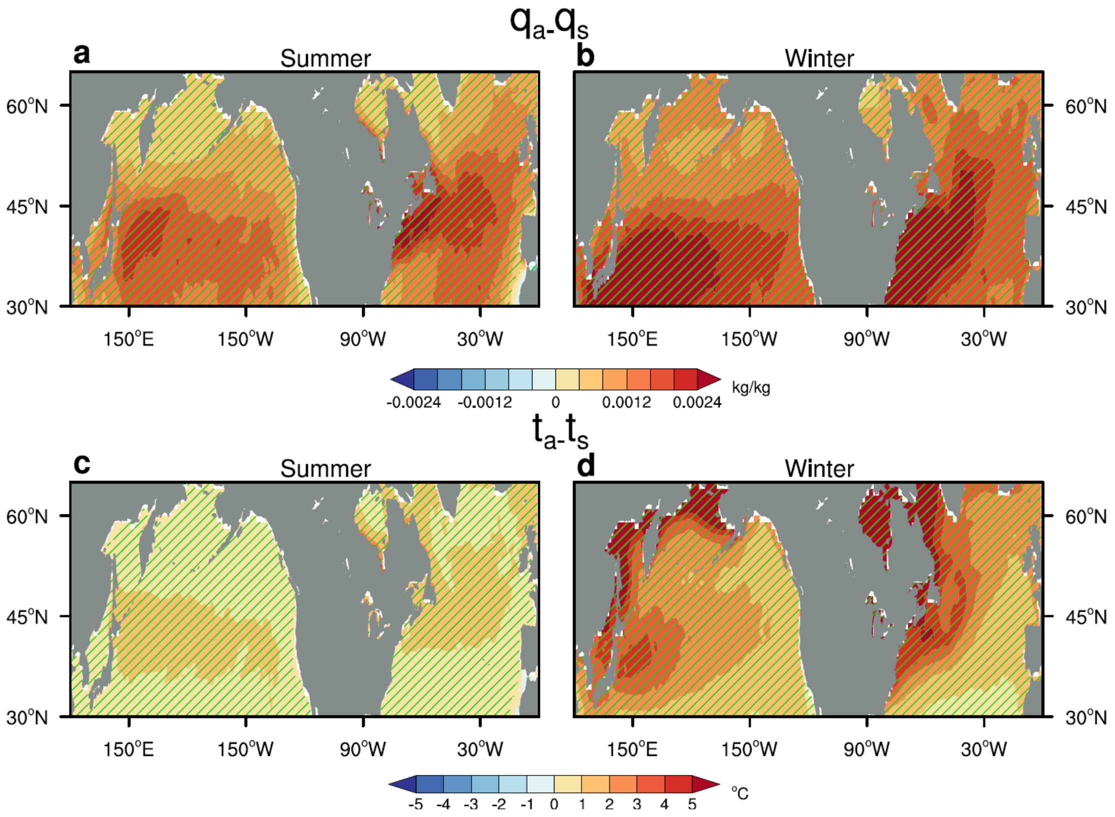

Supplementary Fig. 15. Atmospheric river (AR)-related air-sea humidity and temperature contrasts. Composites of AR-related anomalies in air–sea specific humidity difference (kg/kg) during **(a)** summer and **(b)** winter AR days. **(c–d)** Same as **(a–b)**, but for air–sea temperature difference anomalies (°C). Hatched regions indicate areas where composite anomalies are statistically significant at the 90% confidence level.

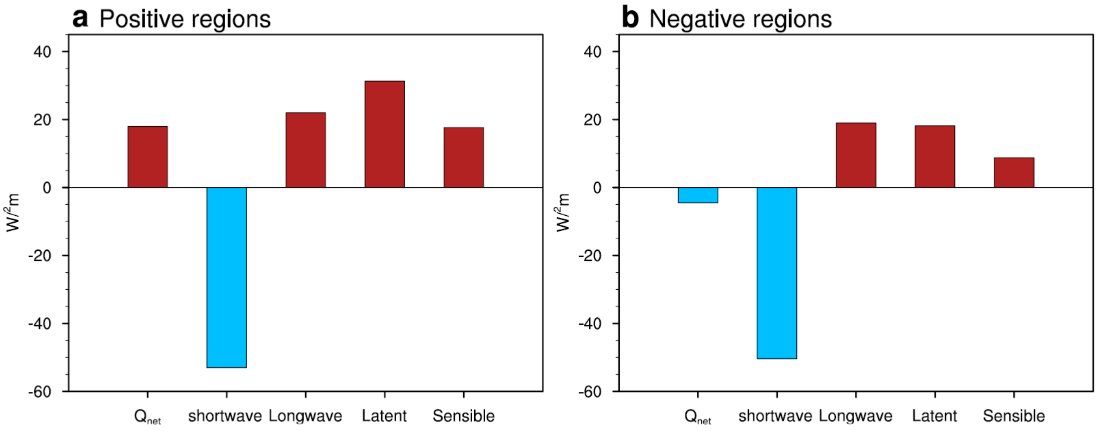

Supplementary Fig. 16. Decomposition of net surface heat flux (Q_{net}) anomalies on atmospheric river (AR) days. Composites of Q_{net}, net surface shortwave radiation, net surface longwave radiation, latent heat flux, and sensible heat flux anomalies (W/m^2) during summer AR days in the positive **(a)** and negative **(b)** regions of sea surface temperature (SST) tendency.

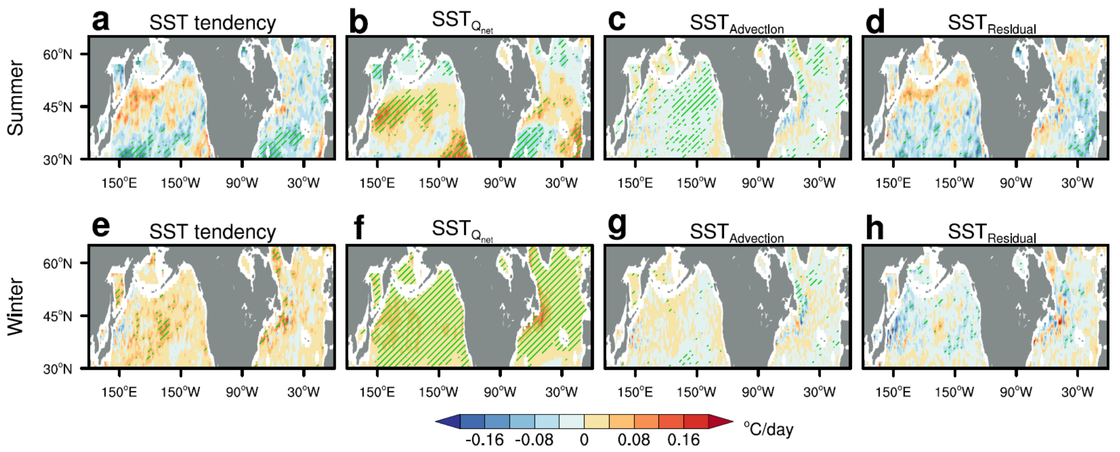

Supplementary Fig. 17. Spatial decomposition of sea surface temperature (SST) tendency based on ocean mixed-layer heat budget analysis during atmospheric river (AR) days. (a–d) Composite SST tendency (°C/day) and the contributions from net surface heat flux ($SST_{Q_{net}}$), horizontal oceanic advection($SST_{Advection}$), and the residual term ($SST_{residual}$) during summer AR days over the period 2019–2023. **(e–h)** Same as **(a–d)**, but for winter AR days. Hatched regions indicate areas where composite anomalies are statistically significant at the 90% confidence level.

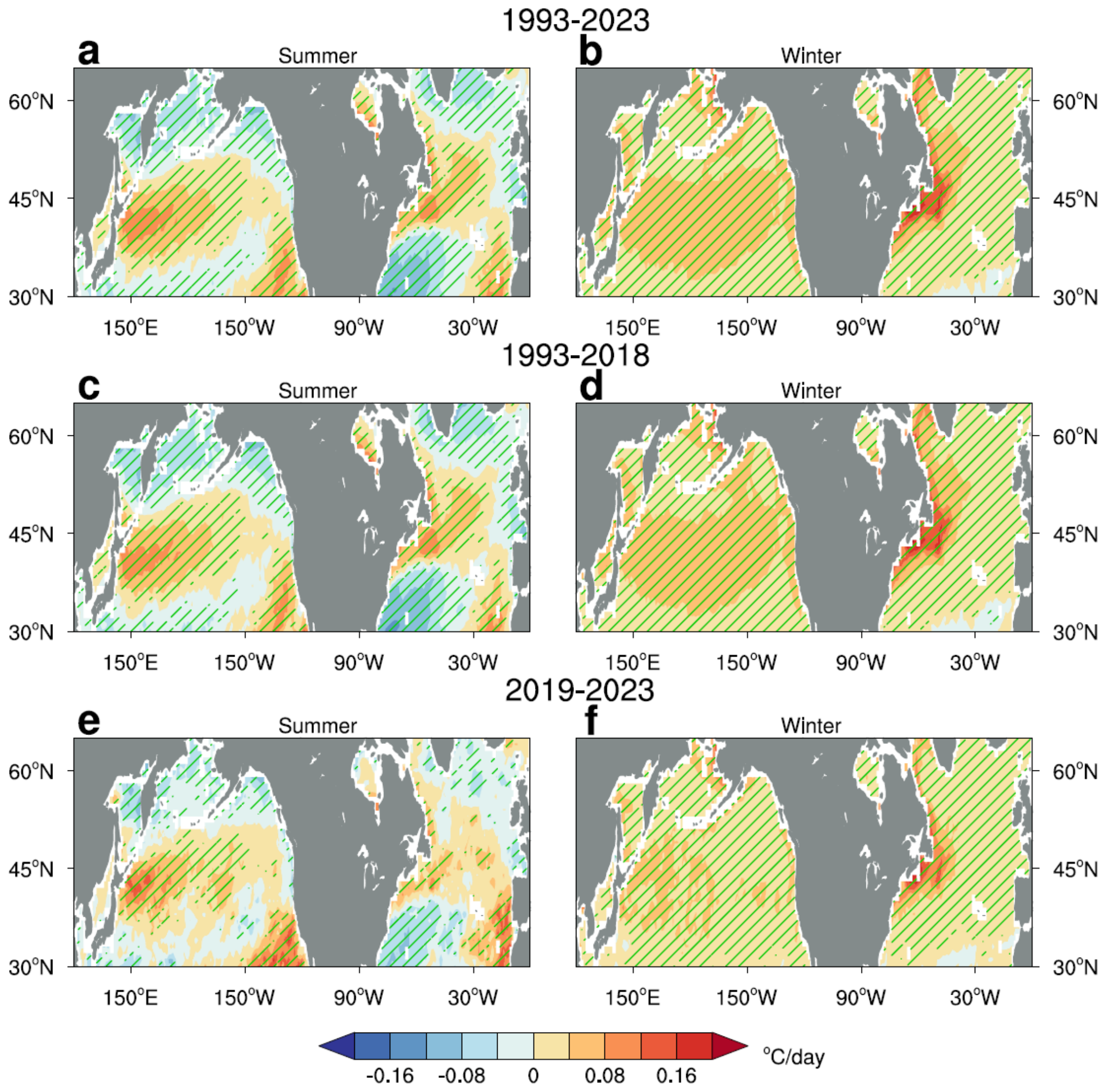

Supplementary Fig. 18. Sea surface temperature (SST) tendency contributed by net surface heat flux (Q_{net}) anomalies during atmospheric river (AR) days. (a–b) Composite SST tendency (°C/day) contributed by Q_{net} anomalies during summer and winter AR days over the period 1993-2023. **(c–d)** Same as **(a–b)**, but for the period 1993-2018. **(e-f)** Same as **(a–b)**, but for the period 2019-2023. Hatched regions indicate areas where composite anomalies are statistically significant at the 90% confidence level.

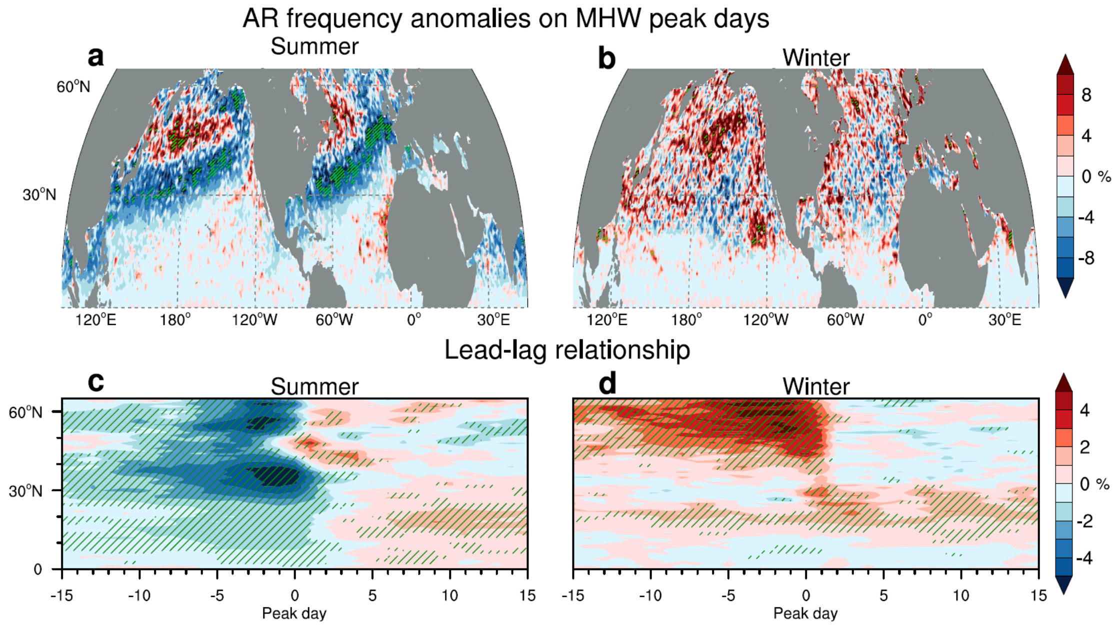

Supplementary Fig. 19. Relationship between atmospheric rivers (ARs) and marine heatwaves (MHWs) based on detrended datasets. (a, b) Composite anomalies of daily AR frequency (%) on MHW peak days during boreal summer (June–August) and winter (December–February). **(c, d)** Composite anomalies of zonal-mean daily AR frequency (%) from 15 days before (−15) to 15 days after (+15) MHW peak days in summer and winter. Hatched regions indicate areas where composite anomalies are statistically significant at the 90% confidence level.

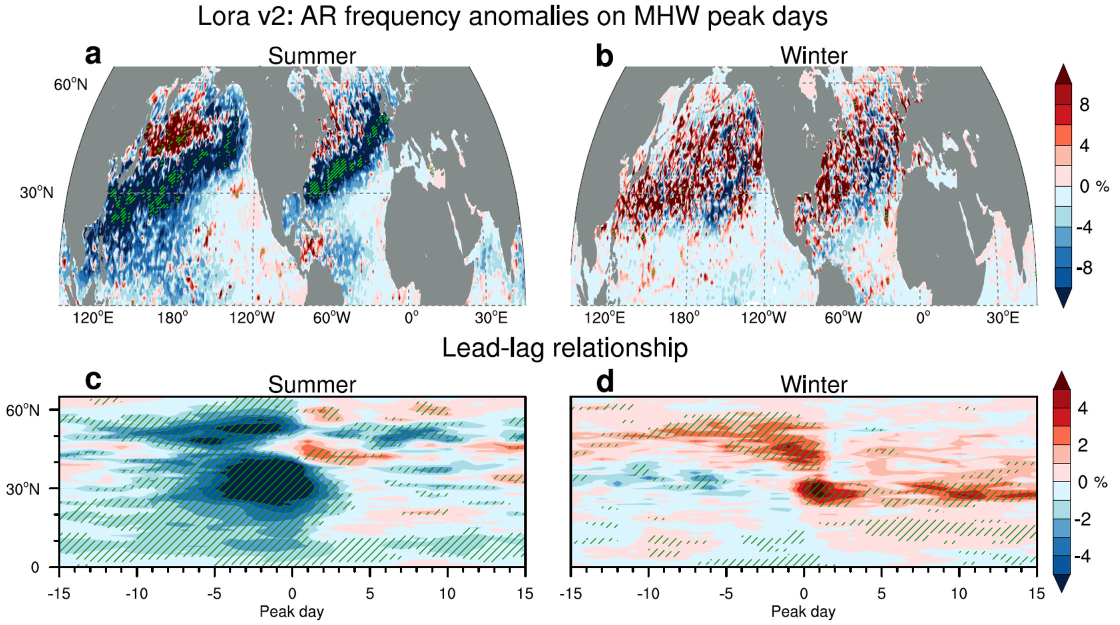

Supplementary Fig. 20. Relationship between atmospheric rivers (ARs) and marine heatwaves (MHWs) based on AR-CONNECT detection algorithm. (a, b) Composite anomalies of daily AR frequency (%) on MHW peak days during boreal summer (June–August) and winter (December–February). **(c, d)** Composite anomalies of zonal-mean daily AR frequency (%) from 15 days before (−15) to 15 days after (+15) MHW peak days in summer and winter. Hatched regions indicate areas where composite anomalies are statistically significant at the 90% confidence level.

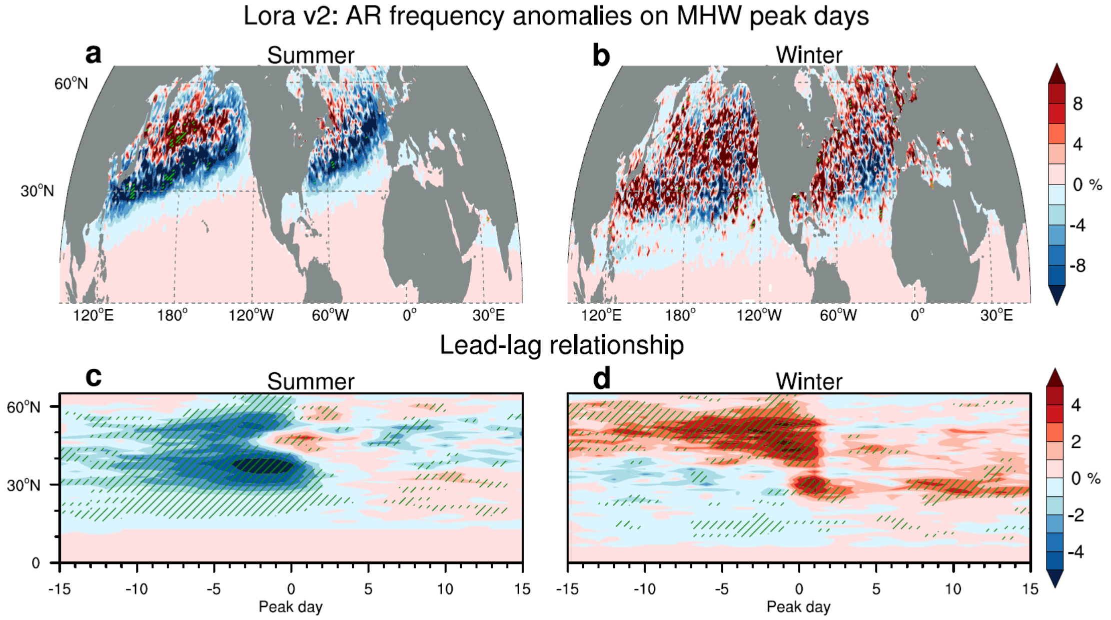

Supplementary Fig. 21. Relationship between atmospheric rivers (ARs) and marine heatwaves (MHWs) based on Lora v2 detection algorithm. (a, b) Composite anomalies of daily AR frequency (%) on MHW peak days during boreal summer (June–August) and winter (December–February). **(c, d)** Composite anomalies of zonal-mean daily AR frequency (%) from 15 days before (−15) to 15 days after (+15) MHW peak days in summer and winter. Hatched regions indicate areas where composite anomalies are statistically significant at the 90% confidence level.
