# Supplementary material for: Detected impacts of atmospheric rivers on marine heatwaves
Source: Nat Commun. 2026 Jun 11;17:7450. doi: 10.1038/s41467-026-74249-9 (PMC13408837; doi:10.1038/s41467-026-74249-9)
Supplement: Supplementary file 2 — Transparent Peer Review file [file 41467_2026_74249_MOESM2_ESM.pdf]

nature portfolio

Peer Review File

Detected Impacts of Atmospheric Rivers on Marine Heatwaves

Corresponding Author: Dr Shineng Hu

This file contains all reviewer reports in order by version, followed by all author rebuttals in order by version.

Attachments originally included by the reviewers as part of their assessment can be found at the end of this file.

Version 0:

Reviewer comments:

Reviewer #1

(Remarks to the Author)

Review of the manuscript “Detected Fingerprints of Atmospheric Rivers on Marine Heatwaves”

In this paper, authors study the role of atmospheric rivers (ARs) in the development of marine heat waves (MHWs) in Northern Pacific and Atlantic Oceans. With 30+ years of reanalysis data, authors composite various analysis selected with reference to MHW peak days. The analysis include atmosphere conditions, surface heat fluxes, SST tendencies, and mixed layer budget. Authors bring attention to how ARs’ increase (decrease) in occurrence prior to MHW peak days in winter (summer). They further demonstrate that SST tendencies enforced by AR are mostly coming from surface fluxes. The budget analysis shows that ARs exert opposite impacts on anomalous shortwave radiation depending on background season. This is a key reason to explain the season-dependent relationship between anomalous AR frequency and SST tendencies. Authors also state that they have confirm similar statistics among 15 selected CMIP6 models.
This is a timely research highlighting the role of AR as a fast weather phenomenon (less than 5 days) that impacts slower MHW events (5+ days) using high-resolution ocean assimilation dataset. However, I find methodology not clear enough if one wants to replicate their results. Furthermore, I am concerned with the absent of significance test / uncertainty in most of the figures presented. I believe if these improvements are made, it can greatly enhance paper’s quality, and make the paper reader-friendly, thus broadening its impact. Therefore, I suggest major revision before considering with publication.

Major comments
Missing details of how mixed layer depth is determined. Because there is a variety of mixed layer algorithms and the mixed layer thickness is important in budget computation, I recommend authors to explain how mixed layer in their dataset is determined.
The budget computation is unclear. Do GLORYS12V1 and CMIP6 models output these terms directly? If yes, authors should state it explicitly. If no, authors should document these details in either the method section or supplementary.
The significant tests seem to be missing in almost all the plots. Because AR is highly variable in time and space, uncertainty estimate is a crucial part of such studies. (Extended Data Figure 10 is meaningful because authors used stippling to show such statistical information)
Have authors tested if their results are sensitive to whether the data (SST or AR IVT) is detrended?

Minor comments
Lines 51—52 “Such spatial collocation (meaning Extended Data Fig 1 a,b) … ”: At the first glance, the collocation is not very obvious to me. While they (AR frequency and MHW days) both have thin tails to the west of the ocean basins, MHW days pattern extends much more southward than AR frequency on the eastern side of the Pacific Ocean. On the Atlantic Ocean, it is the AR frequency that extends more southward than MHW days. Authors may want to expand the sentence to narrow down the exact region of collocation they are referring to.
ARs in winter carry anomalous westerly component, which on the Pacific Ocean should result in advective cooling, as is shown in Hsu et al. (2024) Figure 5c. However, authors’ Figure 2g shows slightly positive SST tendency in most of the domain. Can authors provide some rational as for why AR can induce positive SST trend in the majority of the domain

chosen?
What causes the strong Q_net and SST_residual along the western coastal Atlantic Ocean as shown in Figures 2f and 2h? The role of ocean response seems to be substantial, contradicting the statement in Line 126—128 “The residual term, …, shows a modest contribution.” Maybe some quantitative description can help substantiate the context.
Line 131—132: “The residual term also includes an overall cooling effect … ”, it does not seem to be the case for Pacific Ocean summer, as they seem to be offset by a phase difference of 90 degree comparing Figures 2b and 2d. For the winter, is hard to tell due to very weak absolute values (because the signal over Atlantic Ocean is very large, thus the large range of colorbar). Perhaps authors should consider providing basin-wise pattern correlations.
Physical consistency between GLORYS12V1 and ERA5 is important. Is GLORYS12V1 forced with ERA5 surface fluxes? Authors should include such information when introducing their datasets.
MHWs definition part:
Line 340-341: “11-day window … smoothed with a 31-day moving average…”. This might be very hard to parse for readers. Can authors break it down so it can be easy to follow?
AR definition part:
Lines 352—354: “A grid is tagged as part of an AR ... that is computed with a five-month moving window centered on each calendar month” is ambiguous to me. Does that mean there is a jump of threshold value when the date crosses the month? “Daily AR frequency” needs to be defined.
Line 358—359: “A day is defined as an AR day at a given grid point if more than two AR events are detected.” Is ambiguous to me. For example, does it mean two consecutive AR events with-in 24 hours? Does it count if two events cross 00 hour, such as 6pm~6am?
Figure 1: I am confused by description of (c, d). While zonal mean can be applied to AR frequency, how is MHW peak day determined in this context? Every grid point has its own MHW peak day, what does “zonal mean” here mean?
Figure 3: It took me a few seconds to realize the x-axis is longitude. Authors might consider put “longitude” or any indicator to explicit tell readers what that axis represents.
For Figures 1a, b, Extended Data Figures 1a, b, Extended Data Figures 2a, b, Extended Data Figures 10a, b, it will be more informative if authors label longitudes and latitudes.
The “horseshoe-shaped” pattern over Pacific Ocean seems to be an important feature. While I don’t think it is an easy mechanism, it would be useful and informative to provide readers some speculation.
What is the role of precipitation? While authors focus on the impact of the thermal energy, in the context of MHW, freshwater flux as AR seems to be an important aspect. Is it possible to put in some outlook, or connection?
Reference
Hsu, T. Y., Mazloff, M. R., Gille, S. T., Freilich, M. A., Sun, R., & Cornuelle, B. D. (2024). Response of sea surface temperature to atmospheric rivers. Nature Communications, 15(1), 5018.

Reviewer #2

(Remarks to the Author)
Review of “Detected Fingerprints of Atmospheric Rivers on Marine Heatwaves” by Hu & Hu (2025)

This paper investigates the relationship between atmospheric rivers and marine heatwaves in the North Pacific and North Atlantic. The authors find that marine heatwave events are preceded by anomalously high AR frequency in the boreal winter season, but the reverse is true in boreal summer. This difference is attributed to the different balances of surface energy fluxes during ARs in the summer and winter seasons. To my knowledge, the link between ARs and marine heatwaves has not been previously quantified, making this an original topic.

The paper is well-organized, with clear figures and logical analyses. The text would benefit from a careful proofread, as there are some grammatical errors and unclear phrases, but nothing that cannot be easily fixed. I have a few concerns, primarily, (1) the single AR detection method used, (2) that there is lack of discussion of other large-scale dynamics associated with marine heatwaves, especially in the summer, and, (3) more broadly, the relative importance of boreal winter marine “heatwaves” as defined here. For these reasons, I suggest that this paper undergo major revisions.

Major comments:
#1. The authors employ a percentile-based AR detection algorithm, similar in concept to method employed by Guan & Waliser (2015). This style of detection algorithm is widely used, but has some limitations. In particular, detection algorithms based on local IVT percentiles tend to identify features at the edges of the storm tracks at the expense of storm track cores – since in marginal regions where ARs are less frequent, the IVT threshold is lower, whereas the threshold is higher where AR occur frequently (see Lora et al., 2020). Detection uncertainty remains a significant source of uncertainty when studying atmospheric rivers, particularly the difference between methods using absolute and relative thresholds. In order to better constrain the effects of detection uncertainty, it is best to use multiple AR detection algorithms whenever possible – or at least, one of each broad category (absolute vs relative) – and provide some acknowledgement of the limitations imposed by different methods.

#2. The association found between summertime marine heatwaves and anomalously low AR frequency brings to mind other large-scale drivers of heatwaves, such as atmospheric blocking events – which would presumably lead to both anomalously warm temperatures and reduced AR frequency. Discussion of this link, as well as general discussion of other atmospheric drivers or large-scale processes known to be associated with marine heatwaves, and their association with ARs, would better place the results here in context of the wider literature. Associated with this point, more discussion of why the ‘horseshoe’ shaped pattern is seen in summertime would add value. In particular, what is distinct about the northwestern

portion of basins that leads to the sign change? While this is partially mentioned in the surface flux analysis, more detail about this feature would enrich the discussion.

#3. While I understand that marine heatwaves are generally defined relative to a seasonally-varying threshold, many of the points made in the introduction about the ecological & economic impact of these events seems like they would only pertain to summertime events, which have high absolute temperatures in addition to high temperatures relative to the climatology. Given that ARs seem to be more causally-linked to wintertime events than summertime ones, discussion of this point – as well as more information about the impacts of high wintertime sea temperatures – is needed in the introduction/conclusions.

Line comments.
L18. "suppressed air-sea heat exchanges" – perhaps suppressed cooling would be more accurate? For example, the transfer of sensible heat from the atmosphere to the ocean seems to increase under an AR. Consider rewording.

L19. "spatio-seasonally varying ARs' fingerprints on MHW" – this is unclear, suggest rephrasing. Both in the abstract and title, I am not sure if 'fingerprints' is a clear descriptor.

L38. Language – "featured by," "landfalling precipitation"

L45. Which short-term characteristics of MHW? Please clarify.

L52. Are these events really compound extremes? Over oceans, ARs do not lead to the same sorts of extreme precipitation seen over land.

L54. Again, I am not sure what is meant by 'fingerprints.'

L59. Ocean grid points?

L60. Not clear on the meaning of "spatio-seasonal characteristics"

L69. This is not surprising, as ARs lead to warmer surface temperature anomalies in the winter season cf. the summer. Given the large fraction of wintertime warm temperature extremes that occur within ARs, there is likely a significant overlap between AR days and MHW days in winter.

L148. These surface flux results are largely consistent with those shown globally in Scholz & Lora (2024), and the budget results have similarities to those shown in Hsu et al. (2024) – comparisons to previous literature would enhance the arguments made here.

L151. This figure is interesting, but does not really explain the horseshoe pattern seen in the summer. Also, totaling up the surface flux changes would help the reader better understand the different net effect in each season. Doing the budgets here separately for the positive and negative regions in the Pacific could also provide additional information and clarity.

L166. The background wind speed being stronger in winter is one reason that the turbulent heat fluxes play a larger role, but also consider that the air advected from the lower latitudes into the winter will have a more pronounced temperature/moisture difference regardless, enhancing the turbulent heat fluxes downward.

L170. But why does this not occur in the midlatitude band? See major comment #2.

L176-177. Reducing upward fluxes, perhaps, but it is more accurate to state that they are increasing downward fluxes of sensible heat, in particular. Consider the sign of the net flux on AR days, not just the anomalies.

L182. These CMIP6 results are only mentioned in the discussion, and only very briefly! I suggest including these in the results section, perhaps near the equivalent analyses with reanalysis data.

L192. Are ARs really a 'key driver' of summer MHWs? I can see the argument for wintertime events, but for summertime it seems more likely that the absence of ARs is coincident with other large-scale features such as blocking – see major comment #2. Statements which imply causality, both here and elsewhere, should be more carefully worded.

L194-199. This seems highly speculative, without any citations that show potential examples of these effects.

L203. The poleward shift of ARs is still debated – most recent evidence suggests that it is likely internal variability rather than the forced anthropogenic signal. See Ma et al. (2020), Li & Ding (2024), and Pan et al. (2025) for further discussion.

L205. There is some existing literature about the impact of SSTs on ARs that should be cited here – for example, McClenny et al. (2020).

References
Guan, B. & Waliser, D. E. Detection of atmospheric rivers: Evaluation and application of an algorithm for global studies. Journal of Geophysical Research: Atmospheres 120, 12514–12535 (2015).

Hsu, T.-Y. et al. Response of sea surface temperature to atmospheric rivers. Nat Commun 15, 5018 (2024).
Lora, J. M., Shields, C. A. & Rutz, J. J. Consensus and Disagreement in Atmospheric River Detection: ARTMIP Global Catalogues. Geophysical Research Letters 47, e2020GL089302 (2020).
Scholz, S. R. & Lora, J. M. Atmospheric rivers cause warm winters and extreme heat events. Nature 636, 640–646 (2024).
Ma, W., Chen, G. & Guan, B. Poleward Shift of Atmospheric Rivers in the Southern Hemisphere in Recent Decades. Geophysical Research Letters 47, e2020GL089934 (2020).
Li, Z. & Ding, Q. A global poleward shift of atmospheric rivers. Science Advances 10, eadq0604 (2024).
Pan, M., Hu, S., Zaitchik, B. F. & Pan, W. K. Contrasting historical trends of atmospheric rivers in the Northern Hemisphere. npj Clim Atmos Sci 8, 307 (2025).
McClenny, E. E., Ullrich, P. A. & Grotjahn, R. Sensitivity of Atmospheric River Vapor Transport and Precipitation to Uniform Sea Surface Temperature Increases. Journal of Geophysical Research: Atmospheres 125, e2020JD033421 (2020).

Version 1:

Reviewer comments:

Reviewer #1

(Remarks to the Author)
Authors have made considerable efforts clarifying my previous comments. The only thing I noticed but could not find the answer is that authors seem to have recomputed the data. As a result, the SST cooling of residual term over the western part of Atlantic Ocean has vanished in manuscript Figure 2h. If authors are confident about this change in figure, I think the paper can proceed to publication.

Reviewer #2

(Remarks to the Author)
See attached PDF

[Editorial Note: See end of file]

Version 2:

Reviewer comments:

Reviewer #2

(Remarks to the Author)
The authors have adequately addressed all my concerns. The manuscript is clearer, and the additional analyses have made the arguments more robust. I recommend publication.

Open Access This Peer Review File is licensed under a Creative Commons Attribution 4.0 International License, which permits use, sharing, adaptation, distribution and reproduction in any medium or format, as long as you give appropriate credit to the original author(s) and the source, provide a link to the Creative Commons license, and indicate if changes were made.
In cases where reviewers are anonymous, credit should be given to 'Anonymous Referee' and the source.
The images or other third party material in this Peer Review File are included in the article's Creative Commons license, unless indicated otherwise in a credit line to the material. If material is not included in the article's Creative Commons license and your intended use is not permitted by statutory regulation or exceeds the permitted use, you will need to obtain permission directly from the copyright holder.
To view a copy of this license, visit https://creativecommons.org/licenses/by/4.0/

NCOMMS-25-81021

We would like to thank the editor and the two reviewers for their constructive comments and suggestions. We have addressed the comments and implemented these very useful suggestions in our revised manuscript. Our point-by-point responses are listed below.

Response to Reviewer#1

General Comment

In this paper, authors study the role of atmospheric rivers (ARs) in the development of marine heat waves (MHWs) in Northern Pacific and Atlantic Oceans. With 30+ years of reanalysis data, authors composite various analysis selected with reference to MHW peak days. The analysis include atmosphere conditions, surface heat fluxes, SST tendencies, and mixed layer budget. Authors bring attention to how ARs' increase (decrease) in occurrence prior to MHW peak days in winter (summer). They further demonstrate that SST tendencies enforced by AR are mostly coming from surface fluxes. The budget analysis shows that ARs exert opposite impacts on anomalous shortwave radiation depending on background season. This is a key reason to explain the season-dependent relationship between anomalous AR frequency and SST tendencies. Authors also state that they have confirm similar statistics among 15 selected CMIP6 models.

This is a timely research highlighting the role of AR as a fast weather phenomenon (less than 5 days) that impacts slower MHW events (5+ days) using high-resolution ocean assimilation dataset. However, I find methodology not clear enough if one wants to replicate their results. Furthermore, I am concerned with the absent of significance test / uncertainty in most of the figures presented. I believe if these improvements are made, it can greatly enhance paper's quality, and make the paper reader-friendly, thus broadening its impact. Therefore, I suggest major revision before considering with publication.

Major comments

Comment 1. *Missing details of how mixed layer depth is determined. Because there is a variety of mixed layer algorithms and the mixed layer thickness is important in budget computation, I recommend authors to explain how mixed layer in their dataset is determined.*

Response: We thank the reviewer for this helpful comment. In this study, the mixed layer depth (MLD) is obtained directly from the GLORYS12V1 ocean reanalysis output. In this product, the MLD is defined as the depth at which the density increase relative to the density at 10 m corresponds to a temperature decrease of 0.2 °C under the local surface conditions (as shown in their user manual). We have revised the manuscript to clarify this point in the dataset section (L427-430).

Comment 2. *The budget computation is unclear. Do GLORYS12V1 and CMIP6 models output these terms directly? If yes, authors should state it explicitly. If no, authors should document these details in either the method section or supplementary.*

Response: Thank you for this helpful comment. The heat budget terms analyzed in this study are not directly provided by GLORYS12V1; instead, they are computed diagnostically. Specifically, daily mixed-layer depth, ocean potential temperature, and ocean currents are obtained from the GLORYS12V1 reanalysis, while the net surface heat flux is derived from the ERA5 reanalysis. The horizontal advection term is calculated using daily horizontal currents and horizontal temperature gradients within the mixed layer. The residual term (ε) is estimated as the imbalance of the mixed-layer heat budget. It represents the combined effects of unresolved or implicitly represented processes within the mixed layer, including vertical entrainment, vertical mixing, horizontal diffusion, and other subgrid-scale processes. We have revised the manuscript to clearly document these computational details in the Methods section (L478-486)

Comment 3. *The significant tests seem to be missing in almost all the plots. Because AR is highly variable in time and space, uncertainty estimate is a crucial part of such studies. (Extended Data Figure 10 is meaningful because authors used stippling to show such statistical information)*

Response: We thank the reviewer for this helpful suggestion. In the revised manuscript, we applied statistically significant tests to the composite fields based on two-taild student t test. As shown in Fig. R1, the MHW–AR relationships discussed in this manuscript are statistically significant at the 90% confidence level, indicating that the identified signals are robust. Similarly, we have added the significance tests to most of the figures when we can (Fig. 2, Supplementary Figs. 2,3,4,6,7,8,9,10,11), and updated the figure captions accordingly. We have also described the details of the significant test in the Methods section (L432-433).

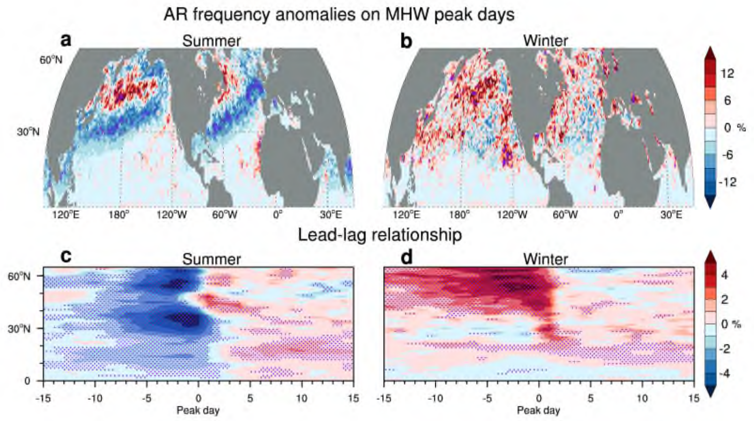

Fig. R1. Relationship between atmospheric rivers (ARs) and marine heatwaves (MHWs). (a, b) Composite anomalies of daily AR frequency (%) on MHW peak days during boreal summer (June–August) and winter (December–February). **(c, d)** Composite anomalies of zonal-mean daily AR frequency (%) from 15 days before (−15) to 15 days after (+15) MHW peak days in summer and winter. Stippling indicates

regions where composite anomalies are statistically significant at the 90% confidence level.

Comment 4. *Have authors tested if their results are sensitive to whether the data (SST or AR IVT) is detrended?*

Response: We thank the reviewer for the comment. We have examined the sensitivity of our results using detrended datasets. The main conclusion remains unchanged: a horseshoe-shaped AR anomaly pattern appears in summer, and a basin-wide positive AR anomaly pattern appears in winter during MHW peaks (Fig. R1 vs. Fig. R2). The related information has been added to the Method section (L433-436).

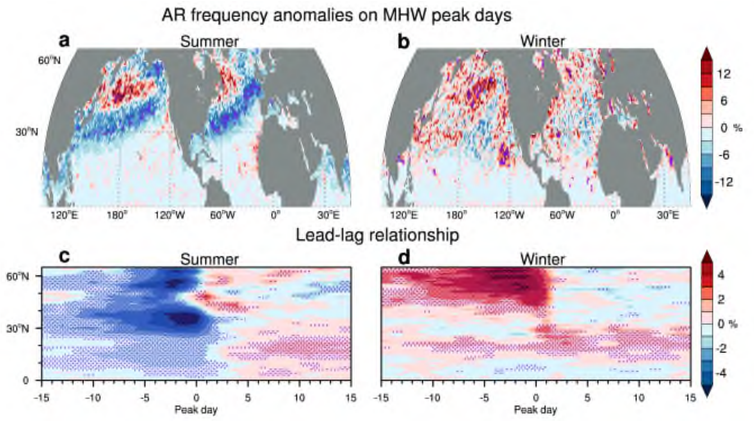

Fig. R2. Relationship between atmospheric rivers (ARs) and marine heatwaves (MHWs) based on detrended datasets. (a, b) Composite anomalies of daily AR frequency (%) on MHW peak days during boreal summer (June–August) and winter (December–February). **(c, d)** Composite anomalies of zonal-mean daily AR frequency (%) from 15 days before (−15) to 15 days after (+15) MHW peak days in summer and winter. Shading indicates regions where composite anomalies are statistically significant at the 95% confidence level based on a Monte Carlo test with 1,000 random resamples.

Minor comments

Comment 1. *Lines 51—52 "Such spatial collocation (meaning Extended Data Fig 1 a,b) … ": At the first glance, the collocation is not very obvious to me. While they (AR frequency and MHW days) both have thin tails to the west of the ocean basins, MHW days pattern extends much more southward than AR frequency on the eastern side of the Pacific Ocean. On the Atlantic Ocean, it is the AR frequency that extends more southward than MHW days. Authors may want to expand the sentence to narrow down the exact region of collocation they are referring to.*

Response: We thank the reviewer for this helpful comment. Here, by "spatial collocation" we do not intend to imply a detailed one-to-one correspondence between the spatial patterns of AR frequency and MHW days, nor that regions of large AR frequency necessarily coincide with regions of large MHW days. Rather, we use this term in a broader sense to emphasize that both ARs and MHWs are quite active in the extratropical regions of the North Pacific and North Atlantic Oceans, where their large-

scale occurrence overlaps. We have deleted the phrase "spatial collocation" to avoid overstating the similarity in their detailed spatial distributions (L56).

Comment 2. *ARs in winter carry anomalous westerly component, which on the Pacific Ocean should result in advective cooling, as is shown in Hsu et al. (2024) Figure 5c. However, authors' Figure 2g shows slightly positive SST tendency in most of the domain. Can authors provide some rational as for why AR can induce positive SST trend in the majority of the domain chosen?*

Response: We thank the reviewer for the insightful comment. We agree that anomalous westerly wind associated with winter ARs should, in principle, induce anomalous southward Ekman transport (i.e., a cooling effect), as shown in Hsu et al. (2024, their Fig. 5c).

To better understand the slightly positive SST tendency shown in our Fig. 2g, we examine the horizontal advection terms using the same units as Hsu et al. (2024). As shown in Fig. R3, the total horizontal oceanic advection anomaly is decomposed into zonal and meridional components (uotx and voty). The meridional advection term (voty) leads to a warming tendency north of 40°N and a cooling tendency in 30°N-40°N (Fig. R3c). This pattern is expected from the anomalous Ekman transport associated with the surface wind anomalies, which exhibit easterlies north of 40°N and westerlies in 30°N-40°N, respectively (Fig. R4). To directly compare our results with Hsu et al. (2024), we repeated the analysis above using their definition of winter season (October–March) based on both GlORYS12V1 and ECCOV4r4 datasets (Fig. R5 and R6). Total horizontal advection produces a cooling tendency over most regions, but minor differences do exist between the two datasets. These results suggest that differences in seasonal definition and datasets can both contribute to the discrepancy between our results and those of Hsu et al. (2024).

We also want to note that horizontal advection contributes only a very small fraction of the total SST tendency compared to net surface heat flux terms. Therefore, the role of horizontal advection is not emphasized in our manuscript.

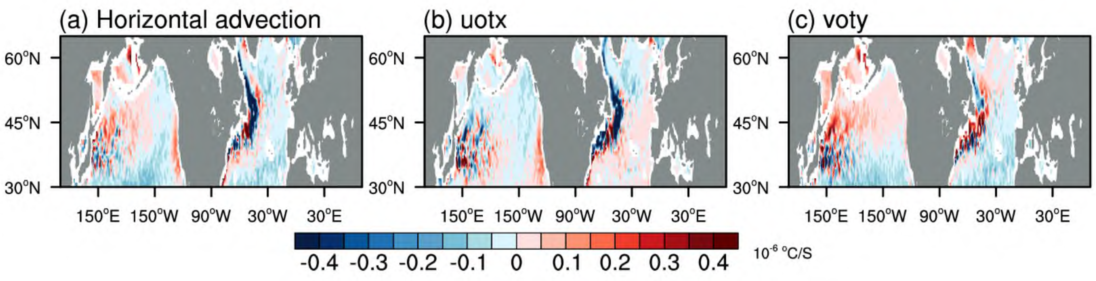

Fig. R3. Composite anomalies (10^{-6} °C/S) of horizontal oceanic advection (a), its zonal component (b), and its meridional component (c) on winter (Dec-Jan) AR days.

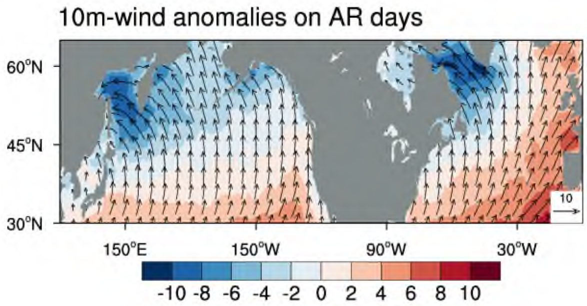

Fig. R4. Composite 10 m-wind anomalies (vector; m/s) and their zonal component (shading; m/s) on winter AR days.

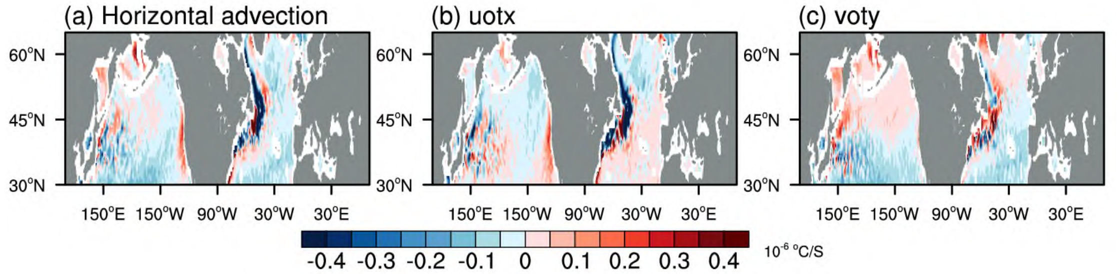

Fig. R5. Composite anomalies (10^{-6} °C/S) of horizontal oceanic advection (a), its zonal component (b), and its meridional component (c) on winter (Oct-Mar) AR days using GlORYS12V1.

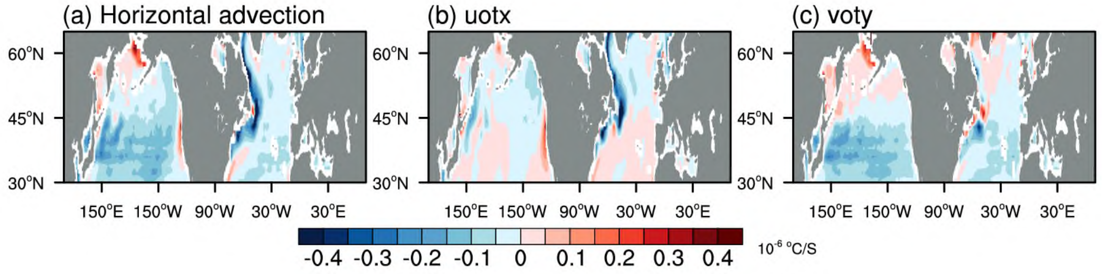

Fig. R6. Composite anomalies (10^{-6} °C/S) of horizontal oceanic advection (a), its zonal component (b), and its meridional component (c) on winter (Oct-Mar) AR days using ECCOV4r4.

Comment 3. *What causes the strong $Qnet$ and $SSTresidual$ along the western coastal Atlantic Ocean as shown in Figures 2f and 2h? The role of ocean response seems to be substantial, contradicting the statement in Line 126—128 "The residual term, …, shows a modest contribution." Maybe some quantitative description can help substantiate the context.*

Response: We thank the reviewer for this helpful comment. As shown in Fig. R7, the strong Q_{net} anomaly along the western Atlantic coastal region primarily arises from enhanced turbulent heat fluxes during winter AR events, which account for approximately **89%** of the total Q_{net} anomaly (Fig. R8). The western Atlantic coast overlies the Gulf Stream, where persistently warm SST coexist with relatively cooler and drier overlying air in winter, resulting in large background air–sea temperature and humidity contrasts. Consequently, the climatological turbulent heat fluxes are

substantially stronger in this region than the surroundings (Fig. R9). AR-related anomalies in air–sea temperature and humidity difference are superimposed on this energetic background state and therefore appear particularly pronounced along the Gulf Stream (Fig. R10), inducing an amplified turbulent heat flux response in this region (Figs. R7c and R7d).

To better quantify the role of the residual term, we calculated the spatial correlation between the Q_{net}-induced SST tendency and the SST tendency associated with the residual term during winter. The correlation coefficients are **–0.58** for the full study region, **–0.59** for the North Atlantic, and **–0.59** for the North Pacific, indicating that the residual term generally acts as a damping component, opposing the Q_{net}-driven SST tendency and modulating the basin-scale spatial pattern.

We further compare the damping rate of the residual term relative to the Q_{net} contribution across different regions, including the western North Atlantic coastal region and the remaining study regions (Fig. R11). For each region, we quantify the contributions of Q_{net} and the residual term to the SST tendency during winter AR days. Along the western coastal region, the Q_{net} -induced warming is approximately **0.059 °C/day**, while the residual term contributes a cooling of about **–0.021 °C/day**. Outside this region, the corresponding values are **0.034 °C/day** and **–0.014 °C/day**, respectively. These results indicate that enhanced downward Q_{net} provides the primary warming contribution along the western Atlantic coast, whereas the residual term acts as a damping mechanism that offsets approximately **one-third** of the Q_{net}-driven warming both inside and outside the western North Atlantic coastal region. We have added the related information in the revised manuscript (L147-157).

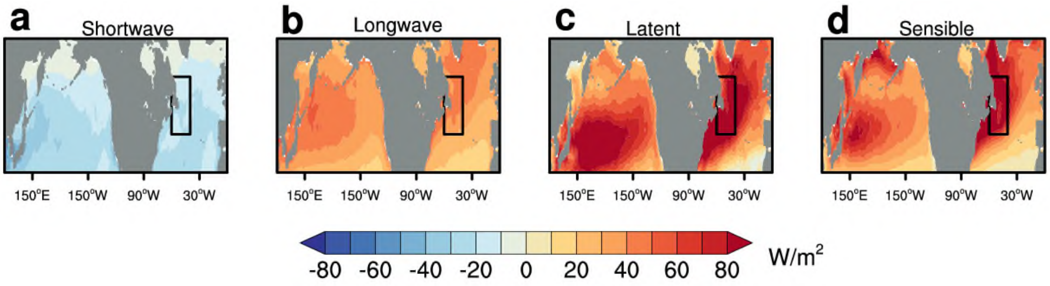

Fig. R7. Decomposition of Q_{net} anomalies on winter AR days. (a–d) Composite anomalies (W/m²) of net shortwave radiation, net longwave radiation, latent heat flux, and sensible heat flux during winter AR days. The black box is used to represent the western Atlantic coastal region.

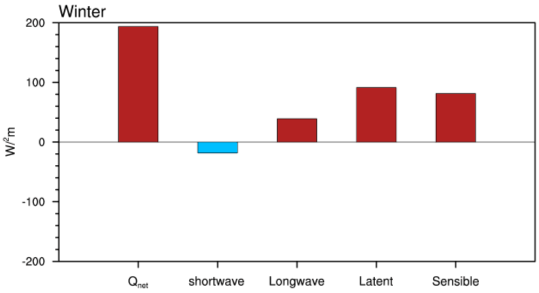

Fig. R8. Composites of Q_{net}, surface net shortwave radiation, net longwave radiation,

latent heat flux, and sensible heat flux anomalies (W/m^2) in the western Atlantic coast during winter AR days.

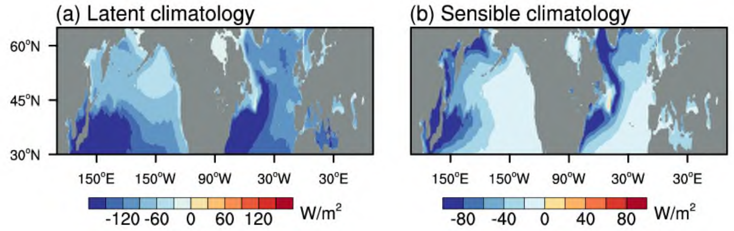

Fig. R9. Climatology of latent heat flux and sensible heat flux anomalies (W/m^2) during winter.

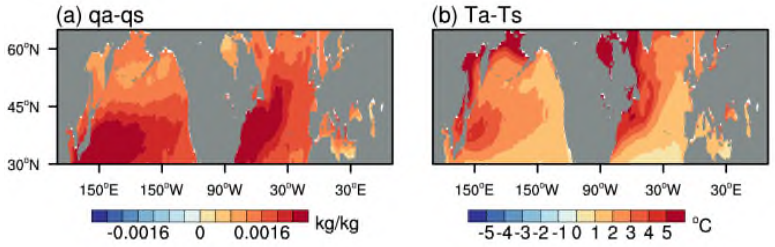

Fig. R10. Composites of air-sea humidity (kg/kg) and air-sea temperature (°C) difference anomalies during winter AR days.

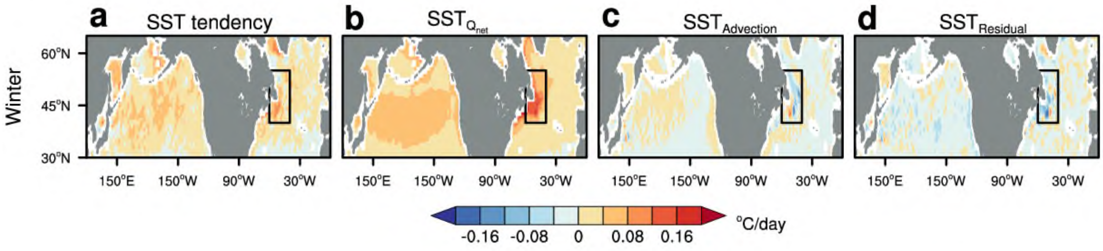

Fig. R11. (a–d) Composite SST tendency (°C/day) and the contributions from Q_{net}, horizontal oceanic advection, and the residual term during winter AR days over the period 1993–2023.

Comment 4. *Line 131—132: "The residual term also includes an overall cooling effect … ", it does not seem to be the case for Pacific Ocean summer, as they seem to be offset by a phase difference of 90 degree comparing Figures 2b and 2d. For the winter, is hard to tell due to very weak absolute values (because the signal over Atlantic Ocean is very large, thus the large range of colorbar). Perhaps authors should consider providing basin-wise pattern correlations.*

Response: We thank the reviewer for these helpful suggestions. To better quantify the damping rate of the residual term, we calculated basin-wide spatial pattern correlations between the Q_{net}-induced SST tendency and the SST tendency associated with the residual term. During winter, the spatial correlation coefficients are **–0.58** for the full study region, **–0.59** for the North Atlantic, and **–0.59** for the North Pacific. These strong, negative correlations indicate that the residual term consistently acts as a damping component in shaping the basin-scale spatial pattern of the total SST tendency in winter. During summer, the spatial correlation coefficients are overall weaker, **–0.33** for the

full study region, **–0.33** for the North Atlantic, and **–0.32** for the North Pacific. The residual term is also positively correlated with summertime mixed-layer depth (MLD) anomalies (r = 0.57), suggesting that regions with a deeper (shallower) ocean mixed layer tend to experience weaker (stronger) residual cooling. Such behavior is physically consistent with vertical mixing processes: when the mixed layer is shallow, the SST is more sensitive to mixing-induced cooling, leading to an enhanced cooling. To avoid overstating the role of the residual term, we have revised the text in L147-157 to clarify that the residual term represents an overall damping process on Q_{net}, rather than a uniform cooling effect, and it is partially modulated by the MLD especially during summer when the climatological MLD is relatively shallow.

Comment 5. *Physical consistency between GLORYS12V1 and ERA5 is important. Is GLORYS12V1 forced with ERA5 surface fluxes? Authors should include such information when introducing their datasets.*

Response: We thank the reviewer for this helpful suggestion. GLORYS12V1 is based on the operational global forecasting system of the Copernicus Marine Environment Monitoring Service (CMEMS). Its ocean model component is the NEMO platform, which is forced at the surface by ECMWF ERA-Interim reanalysis for earlier years and by ERA5 reanalysis since January 1st, 2019. To further assess the physical consistency of combining ERA5 surface heat fluxes with GLORYS12V1, we examined the Q_{net}-induced SST tendency before and after 2019. The spatial patterns and seasonal characteristics during the two periods are overall very similar (Fig. R12). Therefore, ERA5 is used consistently in this study to derive surface heat fluxes and AR-related atmospheric variables across the full analysis period, ensuring a uniform atmospheric reference framework. We have included this information in the dataset description (L415-424).

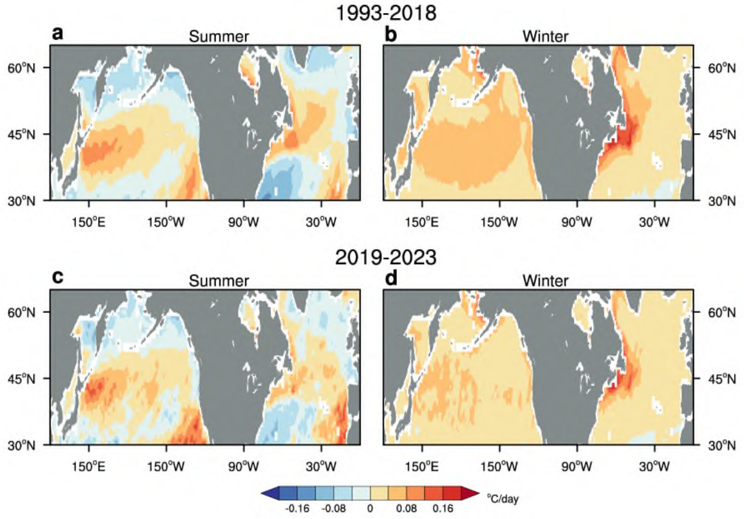

Fig. R12. (a–b) Composite SST tendency (°C/day) contributed by Q_{net} anomalies during summer and winter AR days over the period 1993–2018. (c–d) Same as (a–b), but for the period 2019–2023.

Comment 6. *MHWs definition part: Line 340-341: "11-day window … smoothed with a 31-day moving average…". This might be very hard to parse for readers. Can authors break it down so it can be easy to follow?*

Response: We thank the reviewer for this helpful comment. We have revised the relevant description to improve clarity (L440-443). The revised text now explains the procedure in a step-by-step manner, explicitly describing (i) the use of an 11-day window centered on each calendar day to estimate the 90th percentile SST threshold and (ii) the subsequent application of a 31-day moving average to smooth the threshold and ensure temporal continuity.

Comment 7*. AR definition part:*

1. *Lines 352—354: "A grid is tagged as part of an AR ... that is computed with a five-month moving window centered on each calendar month" is ambiguous to me. Does that mean there is a jump of threshold value when the date crosses the month?*
2. *"Daily AR frequency" needs to be defined.*
3. *Line 358—359: "A day is defined as an AR day at a given grid point if more than two AR events are detected." Is ambiguous to me. For example, does it mean two consecutive AR events with-in 24 hours? Does it count if two events cross 00 hour, such as 6pm~6am?*

Response: We thank the reviewer for these helpful comments. We have revised the manuscript to clarify all related descriptions as detailed below.

(i) The IVT threshold is defined separately for each calendar month and therefore differs among months. However, the five-month moving window is used to construct the climatological sample from which each month-specific threshold is calculated (i.e., the target month together with the two preceding and two following months). Because adjacent months share most of the same data in this window, the resulting month-to-month variation in the threshold is smooth and does not involve abrupt jumps at month boundaries.

(ii) Given the 6-hourly temporal resolution of the ERA5 data, up to four AR detections may occur per day (00, 06, 12, and 18 UTC). Daily AR frequency is defined as the fraction of these 6-hourly time steps during which AR conditions are detected at each grid cell.

(iii) An "AR event" refers to an individual AR detection at a given grid point at one 6-hourly time step. A calendar day is defined as an AR day at a given grid point if AR conditions are detected in more than two of the four 6-hourly time steps within that day, regardless of whether these detections are consecutive. AR detections that span across 00 UTC are counted toward their respective calendar days rather than being treated as a single continuous event.

We have revised the manuscript text to explicitly clarify these definitions and remove potential ambiguities (L459-465).

Comment 8. *Figure 1: I am confused by description of (c, d). While zonal mean can be applied to AR frequency, how is MHW peak day determined in this context? Every grid point has its own MHW peak day, what does "zonal mean" here mean?*

Response: We thank the reviewer for these helpful comments. The peak day of each MHW event is defined as the day on which SST reaches its maximum during that event. In this analysis, MHW peak days are identified independently at each grid point. For the zonal-mean representations shown in Fig. 1c,d, we firstly compute the composites of daily AR frequency anomalies during the 15 days before and after the locally defined MHW peak days at each grid point (following the same compositing approach as in Fig. 1a,b). These gridded composite anomalies are then averaged zonally within each latitude band. In other words, the zonal mean reflects the latitude-dependent mean response of AR frequency to locally defined MHW peak day. We have revised the figure caption and corresponding text to clarify this procedure and avoid confusion (L84-87).

Comment 9. *Figure 3: It took me a few seconds to realize the x-axis is longitude. Authors might consider put "longitude" or any indicator to explicit tell readers what that axis represents.*

Response: We thank the reviewer for this helpful suggestion. To improve clarity, we have explicitly labeled the horizontal and vertical axes as "longitude" and "latitude" in Fig. 3 of the revised manuscript.

Comment 10. *For Figures 1a, b, Extended Data Figures 1a, b, Extended Data Figures 2a, b, Extended Data Figures 10a, b, it will be more informative if authors label longitudes and latitudes.*

Response: Thank the reviewer for these suggestion. We have now added latitude and longitude labels to these figures.

Comment 11. *The "horseshoe-shaped" pattern over Pacific Ocean seems to be an important feature. While I don't think it is an easy mechanism, it would be useful and informative to provide readers some speculation.*

Response: We thank the reviewer for this good suggestion. As also suggested by Reviewer #2 (Comment 10), we conducted further analysis to better understand this horseshoe-shaped pattern in summer. Specifically, we decomposed the Q_{net} anomalies over the positive and negative SST tendency regions shown in Fig. R13 to clarify the regional differences. In both regions, AR events are associated with reduced net downward shortwave radiative flux (cooling) and enhanced downward longwave radiative flux, as well as reduced upward turbulent heat flux anomalies (Fig. R14). However, the relative magnitudes and regional balance among these components differ substantially between the two regions.

As shown in Fig. R14, the shortwave and longwave radiative flux anomalies associated with AR events are comparable between the two areas. In contrast, turbulent heat flux anomalies (including both sensible and latent components) are much stronger in the midlatitude positive SST tendency band, approximately twice those in the negative SST tendency region, which is in turn due mainly to the enhanced air-sea temperature and humidity contrast anomalies (Fig. R15). In the positive SST tendency

region, the enhanced turbulent heat flux response effectively offsets the cooling effect associated with reduced downward shortwave radiation, resulting in a net surface heat gain. In contrast, in the negative SST tendency region, the weaker turbulent heat flux response is insufficient to offset the shortwave-induced cooling, resulting in a net surface heat loss and negative SST tendencies. These differences in the net surface heat flux balance ultimately give rise to the contrasting SST tendencies and explain the observed horseshoe-shaped structure during summer. We have incorporated this additional analysis and interpretation into the revised manuscript (L202-212).

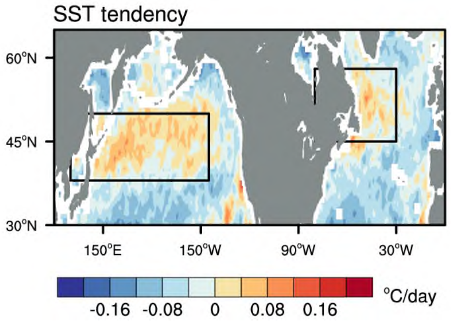

Fig. R13. Composite SST tendency (°C/day) during summer AR days over the period 1993–2023. Black boxes denote the regions of positive SST tendency in the North Pacific and North Atlantic, respectively.

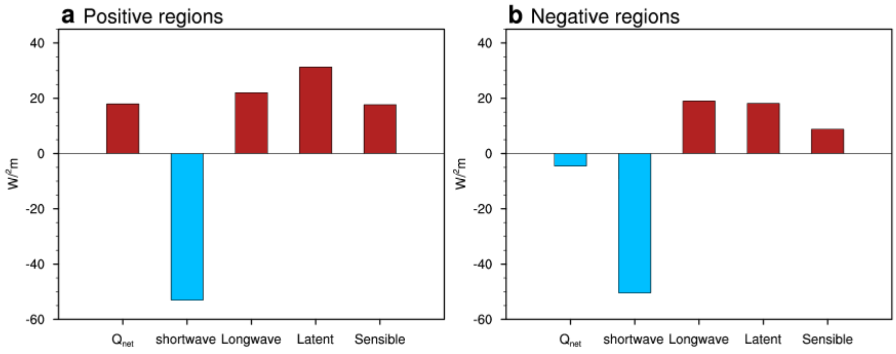

Fig. R14. Composites of Q_{net}, net surface shortwave radiation, net surface longwave radiation, latent heat flux, and sensible heat flux anomalies (W/m^2) during summer AR days in the positive (a) and negative (b) regions of SST tendency.

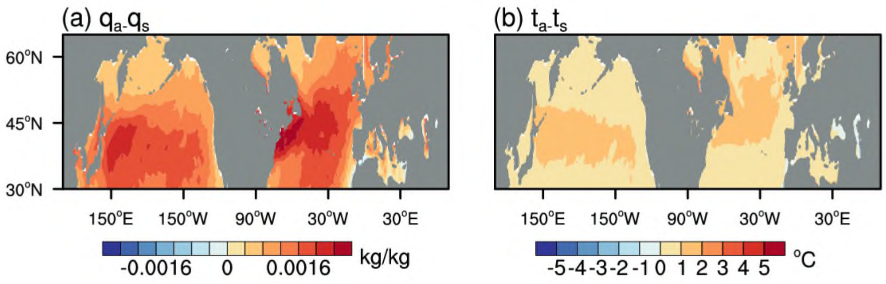

Fig. R15. Composites of AR-related anomalies in (a) air–sea specific humidity difference (kg/kg) and (b) temperature difference (°C) during summer.

Comment 12. *What is the role of precipitation? While authors focus on the impact of the thermal energy, in the context of MHW, freshwater flux as AR seems to be an important aspect. Is it possible to put in some outlook, or connection?*

Response: We thank the reviewer for this insightful suggestion. In this study, we emphasize the dominant role of AR-related turbulent heat fluxes and radiative fluxes in shaping the different responses of MHWs to AR events. As noted by the reviewer, AR-related precipitation may also play an important role in the upper ocean. Several previous studies have shown that precipitation-induced freshwater input can affect upper-ocean temperature variability through its impact on ocean stability and mixing processes (Chen et al. 2025; Jacob and Koblinsky 2007). However, isolating the independent contribution of AR-related precipitation on SST variability from observational datasets is challenging. Quantifying this effect will require targeted climate model simulations that warrant future investigations. We have added a discussion of this limitation and future outlook in the revised manuscript (L223-227).

Chen, L., Z. Zhang, W. Zhang, and S. Yang, 2025: The Role of Precipitation in Modulating Upper-Ocean Structure during a Tropical Cyclone, https://doi.org/10.1175/JPO-D-25-0040.1.

Jacob, S. D., and C. J. Koblinsky, 2007: Effects of Precipitation on the Upper-Ocean Response to a Hurricane, https://doi.org/10.1175/MWR3366.1.

Response to Reviewer#2

General Comment

This paper investigates the relationship between atmospheric rivers and marine heatwaves in the North Pacific and North Atlantic. The authors find that marine heatwave events are preceded by anomalously high AR frequency in the boreal winter season, but the reverse is true in boreal summer. This difference is attributed to the different balances of surface energy fluxes during ARs in the summer and winter seasons. To my knowledge, the link between ARs and marine heatwaves has not been previously quantified, making this an original topic.

The paper is well-organized, with clear figures and logical analyses. The text would benefit from a careful proofread, as there are some grammatical errors and unclear phrases, but nothing that cannot be easily fixed. I have a few concerns, primarily, (1) the single AR detection method used, (2) that there is lack of discussion of other large-scale dynamics associated with marine heatwaves, especially in the summer, and, (3) more broadly, the relative importance of boreal winter marine "heatwaves" as defined here. For these reasons, I suggest that this paper undergo major revisions.

Major comments

Comment 1. *The authors employ a percentile-based AR detection algorithm, similar in concept to method employed by Guan & Waliser (2015). This style of detection algorithm is widely used, but has some limitations. In particular, detection algorithms based on local IVT percentiles tend to identify features at the edges of the storm tracks at the expense of storm track cores – since in marginal regions where ARs are less frequent, the IVT threshold is lower, whereas the threshold is higher where AR occur frequently (see Lora et al., 2020). Detection uncertainty remains a significant source of uncertainty when studying atmospheric rivers, particularly the difference between methods using absolute and relative thresholds. In order to better constrain the effects of detection uncertainty, it is best to use multiple AR detection algorithms whenever possible – or at least, one of each broad category (absolute vs relative) – and provide some acknowledgement of the limitations imposed by different methods.*

Response: We thank the reviewer for this constructive suggestion. To better assess the robustness of the AR–MHW relationship to AR detection algorithms, we additionally applied two independent AR detection algorithms: AR-CONNECT (Shearer et al. 2020), which is an object-based tracking algorithm using absolute IVT thresholds, and Lora v2 (Lora et al. 2017; Skinner et al. 2020), a hybrid relative–absolute detection method that accounts for background climate variability. As shown in Figs. R16 and R17, the main conclusions in this study remain unchanged across all three detection methods. Specifically, the horseshoe-shaped AR anomaly pattern in summer and the basin-wide positive AR anomaly pattern in winter are reproduced by these two methods, particularly over the extratropical regions that are the focus of this study. While some quantitative differences in AR anomaly magnitude are present among these algorithms, the overall spatial structures and seasonal contrasts remain unchanged. These results

indicate that the key findings of this study are not sensitive to the choice of AR detection algorithms. We also acknowledge that detection uncertainty may influence the amplitude of diagnosed AR frequency anomalies. We have added a discussion of this methodological sensitivity in the revised manuscript (L466-471).

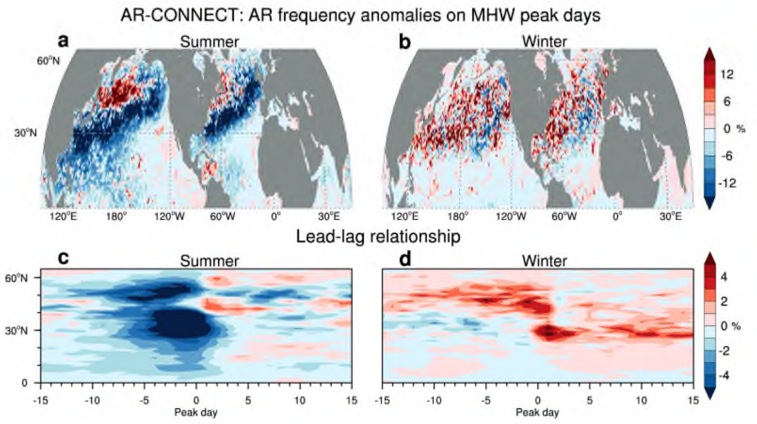

Fig. R16. Relationship between atmospheric rivers (ARs) and marine heatwaves (MHWs) based on AR-CONNECT detection algorithm. (a, b) Composite anomalies of daily AR frequency (%) on MHW peak days during boreal summer (June–August) and winter (December–February). (c, d) Composite anomalies of zonal-mean daily AR frequency (%) from 15 days before (−15) to 15 days after (+15) MHW peak days in summer and winter.

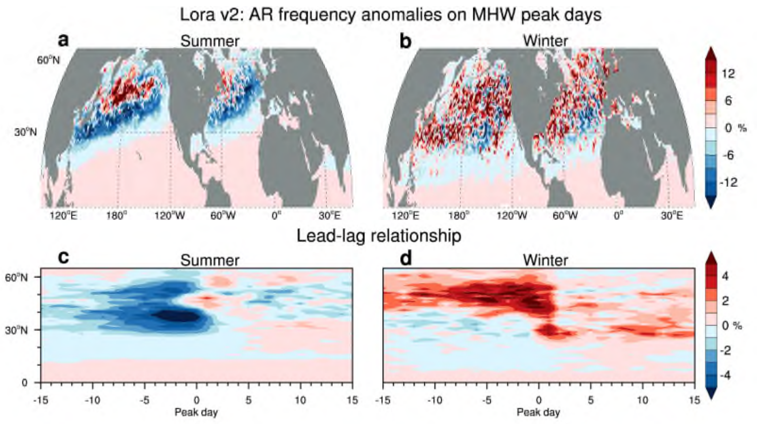

Fig. R17. Relationship between atmospheric rivers (ARs) and marine heatwaves (MHWs) based on Lora v2 detection algorithm. (a, b) Composite anomalies of daily AR frequency (%) on MHW peak days during boreal summer (June–August) and winter (December–February). (c, d) Composite anomalies of zonal-mean daily AR frequency (%) from 15 days before (−15) to 15 days after (+15) MHW peak days in summer and winter.

Lora, J. M., J. L. Mitchell, C. Risi, and A. E. Tripati, 2017: North Pacific atmospheric rivers and their influence on western North America at the Last Glacial Maximum. Geophysical Research Letters, 44, 1051–1059, https://doi.org/10.1002/2016GL071541.

Shearer, E. J., P. Nguyen, S. L. Sellars, B. Analui, B. Kawzenuk, K. Hsu, and S.

Sorooshian, 2020: Examination of Global Midlatitude Atmospheric River Lifecycles Using an Object-Oriented Methodology. Journal of Geophysical Research: Atmospheres, 125, e2020JD033425, https://doi.org/10.1029/2020JD033425.

Skinner, C. B., J. M. Lora, A. E. Payne, and C. J. Poulsen, 2020: Atmospheric river changes shaped mid-latitude hydroclimate since the mid-Holocene. Earth and Planetary Science Letters, 541, 116293, https://doi.org/10.1016/j.epsl.2020.116293.

Comment 2. *The association found between summertime marine heatwaves and anomalously low AR frequency brings to mind other large-scale drivers of heatwaves, such as atmospheric blocking events – which would presumably lead to both anomalously warm temperatures and reduced AR frequency. Discussion of this link, as well as general discussion of other atmospheric drivers or large-scale processes known to be associated with marine heatwaves, and their association with ARs, would better place the results here in context of the wider literature. Associated with this point, more discussion of why the 'horseshoe' shaped pattern is seen in summertime would add value. In particular, what is distinct about the northwestern portion of basins that leads to the sign change? While this is partially mentioned in the surface flux analysis, more detail about this feature would enrich the discussion.*

Response: Thank you for this insightful comment. We agree that ARs and MHWs can both be related to the variability of large-scale atmospheric circulation. In this study, however, we deliberately focus on ARs as an objectively identifiable synoptic-scale weather system, with the goal of isolating their role in modulating air–sea heat exchange and SST extremes. Our intention is not to imply that ARs act independently of blocking or other modes of variability. We have revised the manuscript to clarify this point and to better situate our results within the broader literature on atmospheric blocking or other large-scale circulation variability in L228-230 of our revised manuscript.

Regarding the horseshoe-shaped pattern in summer, we conducted additional analysis (also see response to Comment #10). Specifically, we decomposed the Q_{net} anomalies over the positive and negative SST tendency regions shown in Fig. R18 to clarify the regional differences. In both regions, AR events are associated with reduced net downward shortwave radiative flux (cooling) and enhanced downward longwave radiative flux, as well as reduced upward turbulent heat flux anomalies (Fig. R19). However, the relative magnitudes and regional balance among these components differ substantially between the two regions. As shown in Fig. R19, the shortwave and longwave radiative flux anomalies associated with AR events are comparable between the two areas. In contrast, turbulent heat flux anomalies (including both sensible and latent components) are much stronger in the midlatitude positive SST tendency band, approximately twice those in the negative SST tendency region, which is in turn due mainly to the enhanced air-sea temperature and humidity contrast anomalies (Fig. R20). In the positive SST tendency region, the enhanced turbulent heat flux response effectively offsets the cooling effect associated with reduced downward shortwave

radiation, resulting in a net surface heat gain. In contrast, in the negative SST tendency region, the weaker turbulent heat flux response is insufficient to offset the shortwave-induced cooling, resulting in a net surface heat loss and negative SST tendencies. These differences in the net surface heat flux balance ultimately give rise to the contrasting SST tendencies and explain the observed horseshoe-shaped structure during summer. We have incorporated this additional analysis and interpretation into the revised manuscript (L202-212).

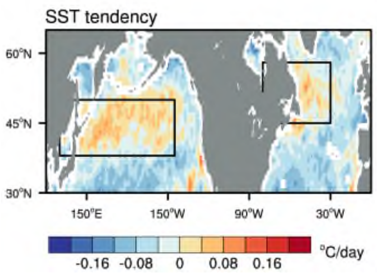

Fig. R18. Composite SST tendency (°C/day) during summer AR days over the period 1993–2023. Black boxes denote the regions of positive SST tendency in the North Pacific and North Atlantic, respectively.

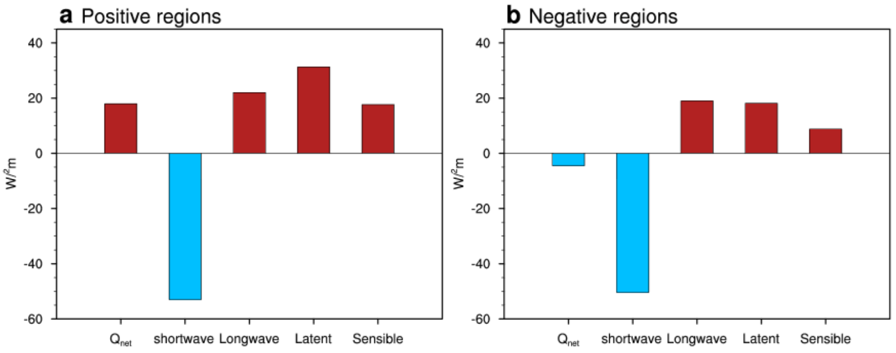

Fig. R19. Composites of Q_{net}, net surface shortwave radiation, net surface longwave radiation, latent heat flux, and sensible heat flux anomalies (W/m^2) during summer AR days in the positive (a) and negative (b) regions of SST tendency.

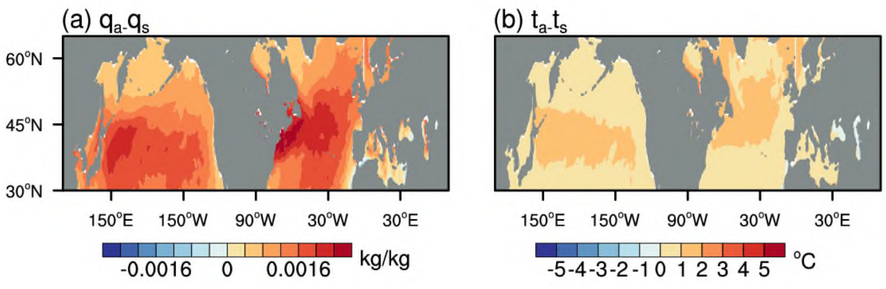

Fig. R20. Composites of AR-related anomalies in (a) air–sea specific humidity difference (kg/kg) and (b) temperature difference (°C) during summer.

Comment 3. *While I understand that marine heatwaves are generally defined relative to a seasonally-varying threshold, many of the points made in the introduction about the ecological & economic impact of these events seems like they would only pertain to summertime events, which have high absolute temperatures in addition to high*

temperatures relative to the climatology. Given that ARs seem to be more causally-linked to wintertime events than summertime ones, discussion of this point – as well as more information about the impacts of high wintertime sea temperatures – is needed in the introduction/conclusions.

Response: We thank the reviewer for this insightful comment. We agree that many of the ecological and economic impacts highlighted in the Introduction are most directly associated with summertime MHWs, when the absolute SST is high in addition to high anomalies relative to the climatology. However, MHWs are more fundamentally defined relative to a seasonally varying climatological threshold and technically can occur anytime throughout the year. It is found that seasonal acclimation to ambient temperatures can lower thermal tolerance, implying that anomalous warming may still have important biological consequences even when absolute temperatures remain moderate in winter (Atkinson et al. 2020; Berkelmans and Willis 1999; Bulger and Tremaine 1985). We have added the related information in L28-33 of the Introduction to clarify this.

In addition to clarifying the role of ARs in wintertime MHW development, our results further highlight the broader importance of AR variability for winter temperature extremes. Specifically, enhanced AR activity suppresses ocean heat loss, favoring the development of positive SST anomalies and therefore MHWs in winter. Conversely, reduced AR frequency is associated with enhanced cold-spell occurrence under a relatively cold climatological background in winter (Fig. R21b and R21d). These findings suggest that ARs act as a key modulator of wintertime air–sea heat exchange, influencing both warm and cold SST extremes.

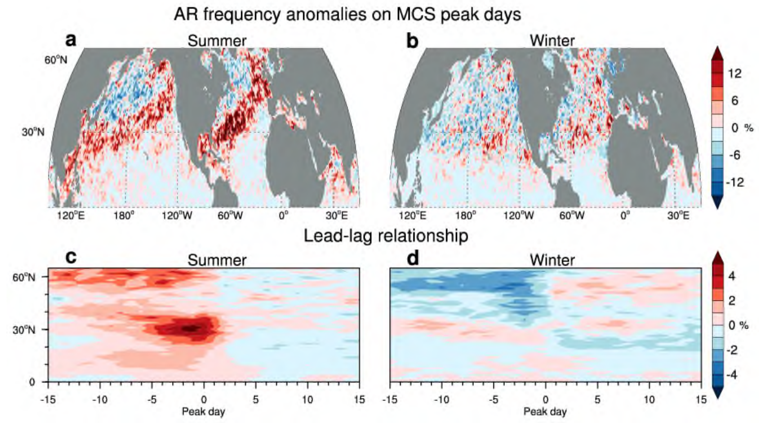

Fig. R21. Relationship between atmospheric rivers (ARs) and marine coldspells (MCSs). (a, b) Composite anomalies of daily AR frequency (%) on MCS peak days during boreal summer (June–August) and winter (December–February). **(c, d)** Composite anomalies of zonal-mean daily AR frequency (%) from 15 days before (−15) to 15 days after (+15) MCS peak days in summer and winter.

Atkinson, J., N. G. King, S. B. Wilmes, and P. J. Moore, 2020: Summer and Winter Marine Heatwaves Favor an Invasive Over Native Seaweeds. Journal of Phycology, 56, 1591–1600, https://doi.org/10.1111/jpy.13051.

Berkelmans, R., and B. L. Willis, 1999: Seasonal and local spatial patterns in the upper thermal limits of corals on the inshore Central Great Barrier Reef. Coral Reefs, 18, 219–228, https://doi.org/10.1007/s003380050186.

Bulger, A. J., and S. C. Tremaine, 1985: Magnitude of Seasonal Effects on Heat Tolerance in Fundulus heteroclitus. Physiological Zoology, 58, 197–204, https://doi.org/10.1086/physzool.58.2.30158567.

Line comments

Comment 1. *L18. "suppressed air-sea heat exchanges" – perhaps suppressed cooling would be more accurate? For example, the transfer of sensible heat from the atmosphere to the ocean seems to increase under an AR. Consider rewording.*

Response: We thank the reviewer for pointing this out. We agree that the phrase "suppressed air–sea heat exchanges" could be misleading. Here, we specifically refer to the reduction of turbulent heat fluxes from the ocean to the atmosphere (including latent and sensible heat fluxes), thereby contributing to ocean warming. We have revised the description in L17 accordingly to improve physical clarity.

Comment 2. *L18. "spatio-seasonally varying ARs' fingerprints on MHW" – this is unclear, suggest rephrasing. Both in the abstract and title, I am not sure if 'fingerprints' is a clear descriptor.*

Response: We thank the reviewer for pointing this out. In the original wording, we intended "fingerprints" to refer to the distinct spatial patterns of AR anomalies associated with summer and winter MHWs. To improve clarity, we have replaced "fingerprints" with "impacts" in both the title and the abstract in the revised manuscript (L1, L19). We have also changed "spatio-seasonally" to "seasonally and regionally" (L19).

Comment 3. *L38. Language – "featured by," "landfalling precipitation"*

Response: We thank the reviewer for pointing this out. We have rephrased the expressions in L43-44 to improve clarity.

Comment 4. *L45. Which short-term characteristics of MHW? Please clarify.*

Response: We thank the reviewer for this helpful comment. Here, by "short-term characteristics," we refer specifically to the development of MHWs on sub-monthly timescales. We have revised the related description in L50 to clarify this point.

Comment 5. *L52. Are these events really compound extremes? Over oceans, ARs do not lead to the same sorts of extreme precipitation seen over land.*

Response: We thank the reviewer for this thoughtful comment. We agree that, over the open ocean, atmospheric rivers do not produce the same types of extreme precipitation impacts as they do over land. In our original wording, the term "compound extremes" was intended to describe the interaction between an extreme oceanic thermal event (a marine heatwave) and an intense atmospheric forcing related to an atmospheric river. To avoid ambiguity and potential confusion, we have revised the manuscript to remove the term "compound extremes" and instead describe these events as interactions

between ARs and MHWs (L57).

Comment 6. *L54. Again, I am not sure what is meant by 'fingerprints.'*
Response: We thank the reviewer for this comment. This concern is closely related to the previous comment regarding the use of the term "fingerprints." As noted above, we have replaced "fingerprints" with "impacts" in the revised manuscript to improve clarity and avoid ambiguity (L58).

Comment 7. *L59. Ocean grid points?*
Response: Corrected (L64).

Comment 8. *L60. Not clear on the meaning of "spatio-seasonal characteristics"*
Response: We thank the reviewer for the good comment. In the original wording, "spatio-seasonal characteristics" was intended to describe the seasonally varying spatial patterns of AR frequency anomalies associated with MHW peak days. To improve clarity, we have revised the text to use more explicit wording in the revised manuscript (L65).

Comment 9. *L69. This is not surprising, as ARs lead to warmer surface temperature anomalies in the winter season cf. the summer. Given the large fraction of wintertime warm temperature extremes that occur within ARs, there is likely a significant overlap between AR days and MHW days in winter.*
Response: We thank the reviewer for these helpful comments. We agree that higher AR frequency anomalies on MHW peak days are, to some extent, expected, given that ARs are associated with warmer surface temperature anomalies in winter. Importantly, however, our subsequent analyses go beyond this statistical overlap by explicitly examining the underlying physical mechanisms. In particular, our mixed-layer heat budget analysis demonstrates that AR-related anomalies in sensible and latent heat fluxes play a dominant role in driving the short-term SST tendencies associated with wintertime MHWs, while shortwave radiation contributes a cooling effect. These results indicate that ARs actively modulate ocean surface warming through reducing turbulent heat fluxes from the ocean to the atmosphere.

Comment 10. *L151. This figure is interesting, but does not really explain the horseshoe pattern seen in the summer. Also, totaling up the surface flux changes would help the reader better understand the different net effect in each season. Doing the budgets here separately for the positive and negative regions in the Pacific could also provide additional information and clarity.*
Response: To better explain the horseshoe-shaped SST tendency pattern observed in summer, we conducted additional diagnostics focusing on the surface heat budget. Specifically, we decomposed the Q_{net} anomalies into their radiative (shortwave and longwave) and turbulent (latent and sensible) components over the positive and negative SST tendency regions shown in Fig. R18. As shown in Fig. R19, during summer, the shortwave and longwave radiative flux anomalies associated with AR events are comparable between the two areas. In contrast, turbulent heat flux anomalies (including both sensible and latent components) are much stronger in the midlatitude positive SST tendency band, approximately twice those in the negative SST tendency

region, which is in turn due mainly to the enhanced air-sea temperature and humidity contrast anomalies (Fig. R20). The enhanced warming effect associated with the turbulent heat flux anomalies acts to offset the cooling effect associated with the shortwave radiative flux anomalies, resulting in a net surface heat gain in the midlatitude band and therefore a horseshoe-shaped pattern in summer. We have included the summed Q_{net} contributions and provided quantitative comparisons of the regional heat budgets in Fig. R22 (Fig. 3 in the revised manuscript).

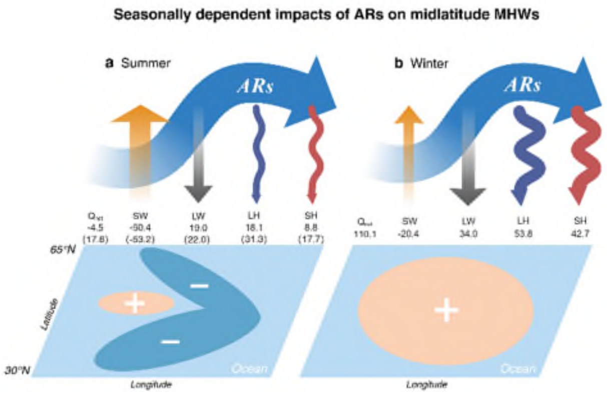

Fig. R22. Mechanistic schematic illustrating the seasonally varying impacts of ARs on MHWs over the North Pacific and North Atlantic (30°–65°N). Distinct seasonal responses are identified in the development of MHWs, with a horseshoe-shaped SST tendency pattern in boreal summer (a) and a basinwide pattern in boreal winter (b). The decomposition of AR-induced Q_{net} anomalies averaged over 30°–65°N is also shown. See text for details of the individual heat flux components (W/m^2). In panel (a), values inside and outside the parentheses correspond to the negative and positive SST tendency regions, respectively.

Comment 11. *L166. The background wind speed being stronger in winter is one reason that the turbulent heat fluxes play a larger role, but also consider that the air advected from the lower latitudes into the winter will have a more pronounced temperature/moisture difference regardless, enhancing the turbulent heat fluxes downward.*

Response: We thank the reviewer for this helpful suggestion. In addition to the stronger background wind speeds in winter, we further examined AR-related anomalies in the air–sea temperature and humidity differences. As shown in Fig. R23, the enhanced role of turbulent heat fluxes in winter is also associated with larger air–sea temperature and humidity contrasts compared to summer. We have added this information in L195-196 of the revised manuscript.

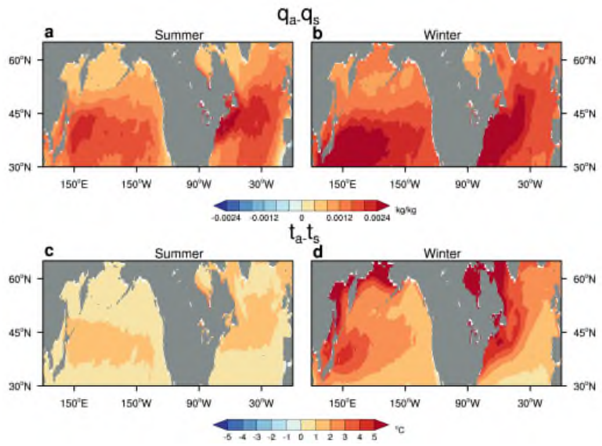

Fig. R23. Composites of AR-related anomalies in air–sea specific humidity difference (kg/kg) during (a) summer and (b) winter AR days. (c–d) Same as (a–b), but for air–sea temperature difference anomalies (°C).

Comment 12. *L170. But why does this not occur in the midlatitude band? See major comment #2.*

Response: We thank the reviewer for this insightful comment. As suggested in Comment#10, we further decomposed the Q_{net} anomalies over the positive and negative SST tendency regions shown in Fig. R18 to better elucidate the regional contrasts. As shown in Fig. R19, during summer, the shortwave and longwave radiative flux anomalies associated with AR events are comparable between the two areas. In contrast, turbulent heat flux anomalies (including both sensible and latent components) are much stronger in the midlatitude positive SST tendency band, approximately twice those in the negative SST tendency region, which is in turn due mainly to the enhanced air-sea temperature and humidity contrast anomalies (Fig. R20). The enhanced warming effect associated with the turbulent heat flux anomalies acts to offset the cooling effect associated with the shortwave radiative flux anomalies, resulting in a net surface heat gain in the midlatitude band and therefore a horseshoe-shaped pattern in summer. We have incorporated this additional analysis and interpretation into the revised manuscript (L202-212).

Comment 13. *L176-177. Reducing upward fluxes, perhaps, but it is more accurate to state that they are increasing downward fluxes of sensible heat, in particular. Consider the sign of the net flux on AR days, not just the anomalies.*

Response: We thank the reviewer for pointing this out. In Fig. R24, we present the climatological mean of Q_{net} and its individual components. The climatological turbulent heat fluxes, including latent and sensible heat fluxes, are predominantly upward over most of the study region (Fig. R24d,e,i,j), indicating a net transfer of heat from the ocean to the atmosphere. During AR days, the advection of warm and moist air leads to enhanced downward latent and sensible heat flux anomalies relative to the climatological mean (Fig. R25d,e,i,j). These downward anomalies reduce the magnitude of the climatological upward turbulent heat fluxes and locally reverse the sign of the net surface heat flux in some regions (Fig. R26d,e,i,j). We have added this information to clarify this point (L163-170, L218).

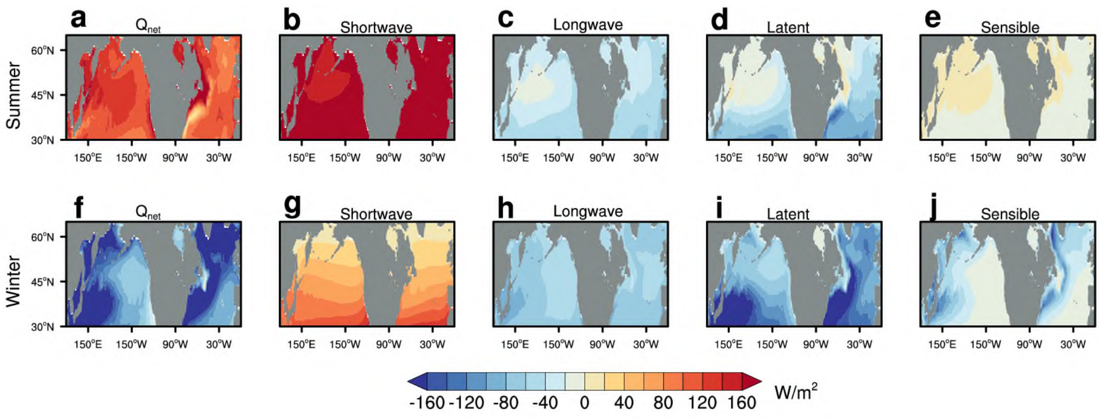

Fig. R24. Decomposition of net surface heat flux climatology. (a–e) Climatology (W/m²) of Q_{net}, shortwave radiation, net longwave radiation, latent heat flux, and sensible heat flux during summer. (f–i) Same as (a-e), but for winter.

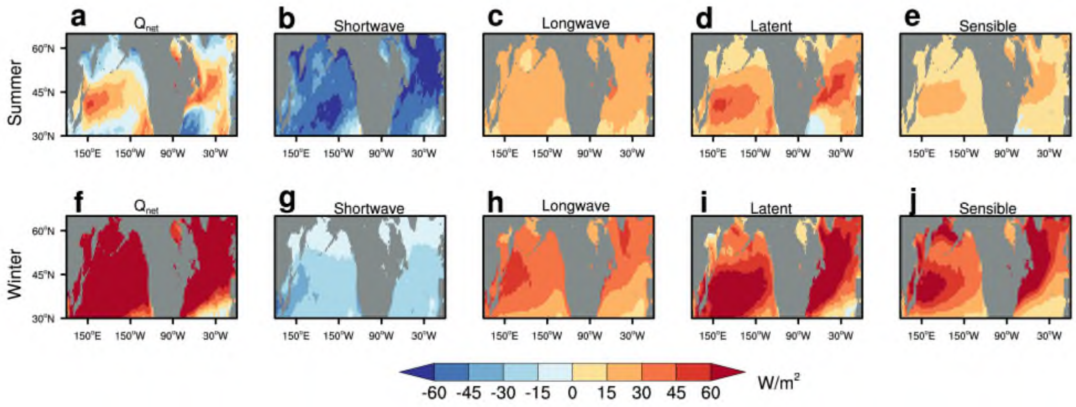

Fig. R25. Decomposition of net surface heat flux anomalies on AR days. (a–e) Composite anomalies (W/m²) of Q_{net}, shortwave radiation, net longwave radiation, latent heat flux, and sensible heat flux during summer AR days. (f–i) Same as (a-e), but for winter AR days.

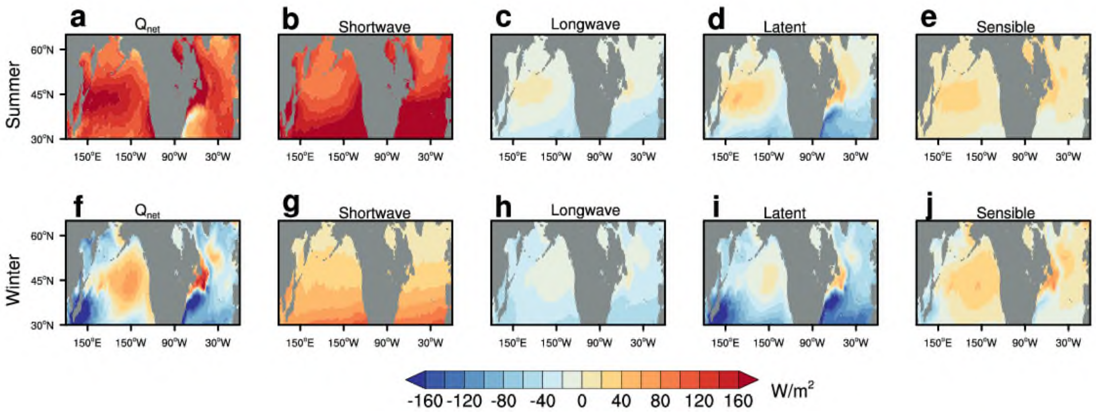

Fig. R26. Decomposition of net surface heat flux on AR days. (a–e) Composite (W/m²) of Q_{net}, shortwave radiation, net longwave radiation, latent heat flux, and sensible heat flux during summer AR days. (f–i) Same as (a-e), but for winter AR days.

Comment 14. *L182. These CMIP6 results are only mentioned in the discussion, and*

only very briefly! I suggest including these in the results section, perhaps near the equivalent analyses with reanalysis data.

Response: We thank the reviewer for this constructive suggestion. We have moved the CMIP6 results from the Discussion to the Results section and placed them alongside the corresponding analyses based on reanalysis data (L74-83; L90-91). The inclusion of the CMIP6 multi-model ensemble is primarily intended to support the robustness of the observed relationship between ARs and MHWs. Based on this verified relationship, we then proceed to explore the associated physical mechanisms using heat-budget analysis, which allows us to diagnose the dominant processes responsible for MHW development during AR events.

Furthermore, we note that the CMIP6 simulations exhibit notable inter-model differences and several intriguing features that may reflect model-dependent representations of air–sea interactions and ocean dynamics. A comprehensive assessment of these inter-model biases and their physical origins would require a dedicated analysis and is therefore beyond the scope of the present study, but it is an important direction for future work.

Comment 15. *L192. Are ARs really a 'key driver' of summer MHWs? I can see the argument for wintertime events, but for summertime it seems more likely that the absence of ARs is coincident with other large-scale features such as blocking – see major comment #2. Statements which imply causality, both here and elsewhere, should be more carefully worded.*

Response: We thank the reviewer for this helpful comment. As discussed in our response to Comment #2, both summertime MHWs and AR frequency anomalies can be related to large-scale atmospheric circulation features such as atmospheric blocking. In this context, AR variability should be interpreted as one pathway through which large-scale circulation anomalies modulate air–sea heat exchange and ocean extremes. To address this concern, we have rephrased this sentence to supplement this point in the Discussion section (L228-230).

Comment 16. *L194-199. This seems highly speculative, without any citations that show potential examples of these effects.*

Response: We thank the reviewer for pointing this out. To address this, we have added relevant references that provide supporting evidence for the potential impacts of MHWs on AR characteristics. Specifically, a recent study focusing on MHWs in the northeastern Pacific showed that ARs following MHW peak days tend to be stronger and shifted northeastward compared to ARs not associated with MHWs (Ma et al. 2025). In addition, case-based studies have indicated that anomalously warm SSTs can enhance moisture convergence along AR pathways (Liu et al. 2021). Together, these previous findings suggest the possibility of two-way interactions between ARs and MHWs. However, we emphasize that quantifying such feedbacks is beyond the scope of the present study and will require targeted investigation using fully coupled atmosphere–ocean models. We have revised the manuscript accordingly to support the discussion with appropriate references (L234-242).

Ma, J., J. Shi, N. J. Holbrook, J. Chen, and Z. Li, 2025: A pathway for Northeast Pacific marine heatwaves to influence temperatures in western Europe during early winter. npj Clim Atmos Sci, 8, 363, https://doi.org/10.1038/s41612-025-01240-4.

Liu, X., and Coauthors, 2021: Ocean fronts and eddies force atmospheric rivers and heavy precipitation in western North America. Nat Commun, 12, 1268, https://doi.org/10.1038/s41467-021-21504-w.

Comment 17. *L203. The poleward shift of ARs is still debated – most recent evidence suggests that it is likely internal variability rather than the forced anthropogenic signal. See Ma et al. (2020), Li & Ding (2024), and Pan et al. (2025) for further discussion.*

Response: We thank the reviewer for this helpful information. We have revised the relevant discussion and added the references in the manuscript to avoid incorrectly interpreting the observed poleward shift as a forced response (L243-249). Specifically, we now emphasize that changes in AR characteristics including their pathways, intensity, and variability may influence the occurrence and intensity of MHWs in the extratropics, regardless of whether these changes arise from internal variability or long-term warming.

Comment 18. *L205. There is some existing literature about the impact of SSTs on ARs that should be cited here – for example, McClenny et al. (2020).*

Response: Thank the reviewer for the very useful information. The related references has been cited in L252 of the revised manuscript.

NCOMMS-25-81021

We would like to thank the editor and the two reviewers for their constructive comments and suggestions. We have addressed the comments and implemented these changes in our revised manuscript. Our point-by-point responses are listed below.

Response to Reviewer#1

General Comment

Authors have made considerable efforts clarifying my previous comments. The only thing I noticed but could not find the answer is that authors seem to have recomputed the data. As a result, the SST cooling of residual term over the western part of Atlantic Ocean has vanished in manuscript Figure 2h. If authors are confident about this change in figure, I think the paper can proceed to publication.

Response: We thank the reviewer for the careful assessment. During our revision process, we identified an error in the calculation of the horizontal advection term related to units conversion. In the revised manuscript, we have corrected that error and recalculated all the terms involved in the heat budget analysis. That explains the small but visible difference in the residual terms (Figs. 2d and 2h) between the revised version and the original version. This updated calculation did not change our main conclusions.

Response to Reviewer#2

This iteration of the paper is improved in clarity, and I appreciate the additional analyses of the 'horseshoe pattern' seen in summertime, which explains a feature that was not well explained in the original manuscript. However, there are a few remaining issues that the authors did not address adequately. Additionally, the methodological clarifications and statistical significance added to plots have raised some new questions.

Major comments

#1. The authors did not provide a response for the following line comment, which still stands. To reiterate, the surface flux results presented in Figure 2 (and supplemental figure 6) are extremely similar to those presented in Scholz & Lora (2024), but the authors do not mention this connection in the relevant portion of the text. The discussion of the sign of the radiative and turbulent heat fluxes is likewise very similar to results from this previous study. Comparison of the budget analyses to results in Hsu et al. (2024) are also not made directly in the text, though some discussion was provided in a response to reviewer #1, who had a similar comment. Not including this previous literature as context risks overstating the originality of results, and does not allow for important differentiation between results here with results from previous studies.

Previous comment: L148. These surface flux results are generally consistent with those shown globally in Scholz & Lora (2024), and the budget results have similarities to those shown in Hsu et al. (2024) – comparisons to previous literature would enhance the arguments made here.

Response: We apologize for missing this comment in our previous response as this comment was accidentally omitted at our editorial stage. We thank the reviewer for pointing out this omission.

In the revised manuscript, we have explicitly acknowledged this connection in the relevant section by adding the following statement (L193-195): "These characteristics are generally consistent with the annual-mean, AR-related surface heat flux anomalies shown by Scholz and Lora (2024), and below we further discuss the distinct features for winter and summer, separately."

In addition, we have expanded the discussion on the heat budget analysis by including a comparison with Hsu et al. (2024) and clarifying the role of Ekman transport in horizontal advection. The revised manuscript includes the following description (L149-156): "Horizontal oceanic advection has only a limited contribution in both seasons (Fig. 2c,g), and its large-scale feature is mainly shaped by the meridional Ekman advection associated with consistent surface wind changes (Supplementary Figs. 6 and 7). More specifically, a westerly (easterly) wind anomaly tends to induce an anomalous southward (northward) Ekman transport and therefore a cooling (warming) effect. Hsu et al.(2024) reported similar features of AR-associated oceanic heat advection in the North Pacific during October-March, and the slight difference of our results comes mainly from the definition of seasons and the ocean reanalysis dataset being used (Supplementary Fig. 8)."

#2. Statistical significance. If the purple stippling on Fig 1 (a) and (b) is the significant regions, it seems a stretch to say that the wintertime pattern is 'basinwide,' especially in the North Atlantic. The significance is stronger in the zonal mean (c, d), but on a point-by-point basis (a, b) it is very sparse in coverage and limited to near the coastlines. The data in this plot is also quite noisy –why is this? Additionally, the purple color makes it very difficult to see the stippling on the map.

Response: We thank the reviewer for this helpful comment. Regarding the visual presentation, we have revised the scheme (from purple stippling to green hatching) to improve the visibility of statistically significant regions across all figures.

The sparse statistical significance in Fig. 1a,b (Fig. R1a,b) mainly reflect the inherently high variability of AR frequency on daily timescales. Also, Fig. 1a and b shows the relationship between two types of extreme events (ARs and MHWs), and the patterns are therefore less spatially coherent (i.e. "noisier") than the AR-induced SST responses in Fig. 2a,e (Fig. R2a, b).

In addition, the composite shown in Fig. 1 is computed for the MHW peak day, whereas the AR influence occurs during the development stage of MHWs, several days prior to the peak. As shown in the zonal-mean lead–lag analysis (Fig. R1c,d), significant AR anomalies tend to emerge during the development stage of MHWs (prior to the peak), indicating that ARs influence the MHW development probably through a cumulative effect prior to the peak of MHW events. To better capture this behavior, we further examined AR frequency anomalies averaged during the five days preceding the MHW peak (Fig. R3). These results indeed show clearer and more spatially coherent spatial structures with an overall higher significance level than the AR frequency

anomalies at the MHW peak, particularly over the North Pacific. This figure and the related discussion have been added in the revised manuscript (Supplementary Fig. 3, L104-109).

We agree with the reviewer that the AR anomaly signal is weaker and less significant in winter than in summer, especially for the North Atlantic. This is consistent with the relatively weak AR-related SST tendency over the North Atlantic in winter (Fig. R2b). In the revised version, we have explicitly clarified this regional contrast (L76-77): "Over the North Atlantic, similar large-scale structures are found for both seasons but overall weaker. Our analysis and discussion below will consider both basins." We have also clarified this point in the corresponding text (L118-120):" Notably, the relatively weak wintertime AR-associated SST change over the North Atlantic is consistent with the weak MHW-associated AR frequency anomaly in this region (Fig. 1b and Fig. 2e)."

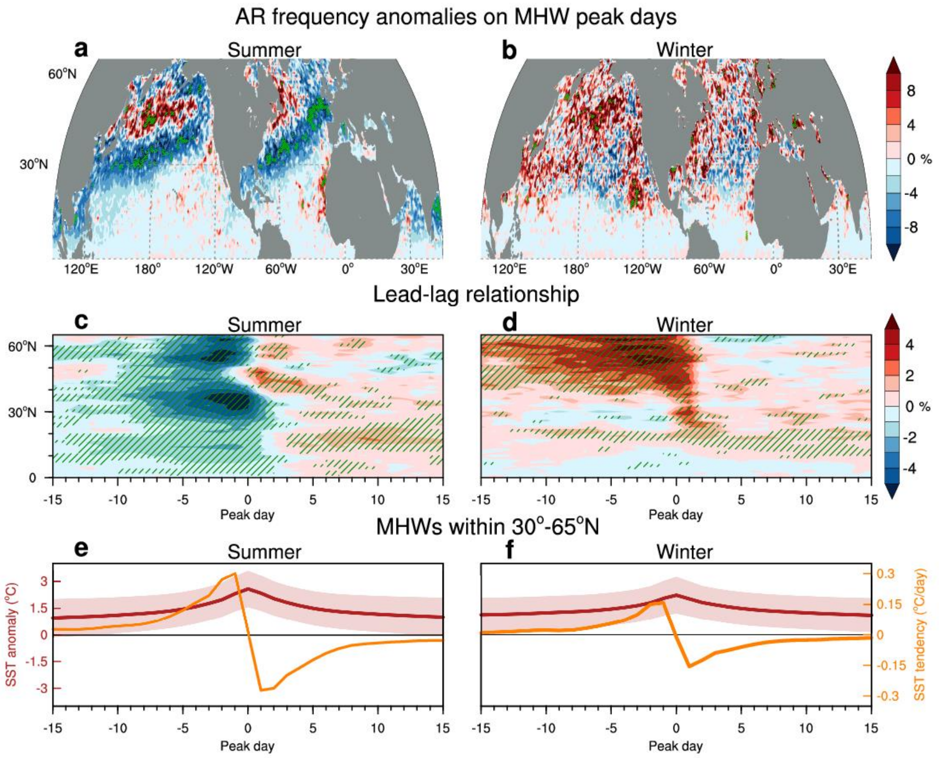

Fig. R1. Relationship between atmospheric rivers (ARs) and marine heatwaves (MHWs). (a, b) Composite anomalies of daily AR frequency (%) on MHW peak days during boreal summer (June–August) and winter (December–February). (c, d) Composite anomalies of zonal-mean daily AR frequency (%) from 15 days before (−15) to 15 days after (+15) MHW peak days in summer and winter. Hatched regions indicate areas where composite anomalies are statistically significant at the 90% confidence level. (e, f) Composite of SST anomalies (°C; red line) and the tendency of SST anomalies (°C/day; orange line) for all MHW events within the 30°N–65°N band, from 15 days before (−15) to 15 days after (+15) MHW peak days in boreal summer and winter. The pink shading indicates the spread (±1 standard deviation) among the MHW events. The analysis period is 1982-2023 for all the panels.

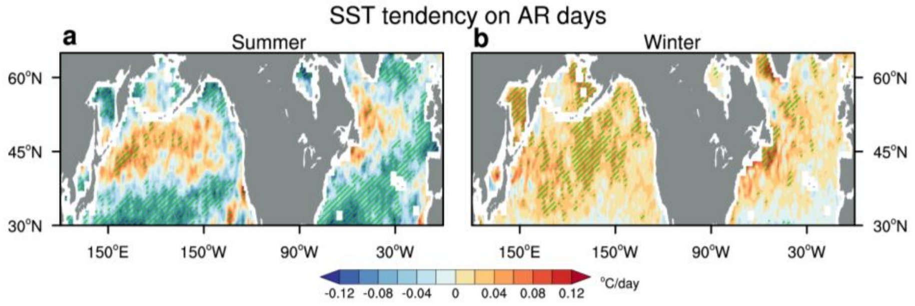

Fig. R2. (a, b) Composite of SST tendency (°C/day) on AR days in boreal summer and winter for the period of 1993-2023. Hatched regions indicate areas where composite anomalies are statistically significant at the 90% confidence level.

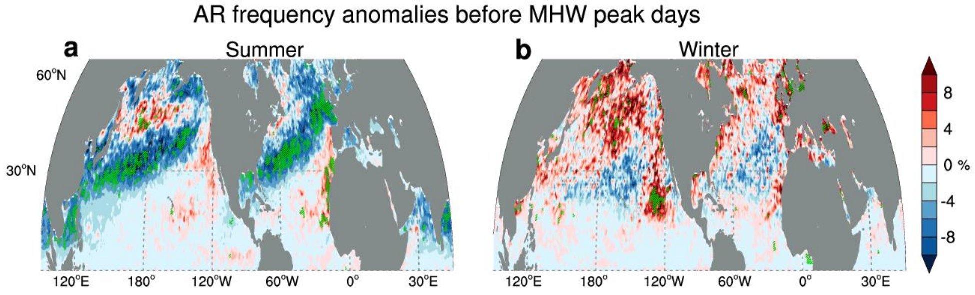

Fig. R3. Composites of AR frequency anomalies averaged over the five days preceding the MHW peak in (a) summer and (b) winter. Hatched regions indicate areas where composite anomalies are statistically significant at the 90% confidence level.

#3. With the new information about the mixed layer budget requested by Reviewer 1, I now have some questions about the interpretation of the residual term. In the original manuscript, I was under the impression that the surface heat flux terms and the advective terms came from the same source. However, since they are from difference sources, it seems tricky to interpret the residual between the two, as it is possible that part of it may be due to differences in data sources. The authors interpret the residual as an 'oceanic damping' of the atmospheric response, because it is largely opposite in sign to the Qnet term. However, this interpretation relies on the consistency of the two data sources. This should be acknowledged.

Response: We agree with the reviewer that the consistency between the atmospheric and ocean reanalysis datasets should be carefully assessed for a better interpretation of the residual term. GLORYS12V1 is based on the operational global forecasting system of the Copernicus Marine Environment Monitoring Service (CMEMS), with its ocean component (NEMO) forced by ECMWF reanalysis products (ERA-Interim before 2018 and ERA5 after 2019). As ERA-Interim has been discontinued and replaced by ERA5, a fully consistent daily heat flux dataset across the entire period is unfortunately not available. Therefore, it is common practice to use ERA5 surface heat fluxes to ensure consistency in the atmospheric forcing fields. However, the concern on the potential inconsistency between the surface heat flux computed from ERA5 (1993-2023) and the surface heat flux used to force GLORYS12V1 (ERA interim during 1993-2018 and ERA5 during 2019-2023) is still valid.

We attempted to address this concern through two additional sensitivity tests. First, we recalculated the ocean mixed layer heat budget for the period of 2019-2023, during which there was no inconsistency issue (i.e., GLORYS12V1 is forced by ERA5). Overall, the results (Fig. R4) are similar to those for the entire period (Fig. 2), but much noisier due to the shorter period and therefore the very small sample size being used. Second, we examined the Q_{net}-induced SST tendency over the entire period, as well as separately before 2018 and after 2019. The spatial patterns and seasonal characteristics are overall very similar for those three periods (Fig. R5). These tests provide us additional confidence in the robustness of our results, although they do not completely eliminate the inherent uncertainty in interpreting the residual term. Therefore, ERA5 is used consistently in this study to derive AR-related surface heat fluxes and atmospheric variables across the full analysis period, ensuring a uniform atmospheric reference framework.

We have now explicitly acknowledged this limitation in the revised manuscript (L156-159). Also, we have added these sensitivity tests to the manuscript with the discussion as follows (L431-442): "We recalculated the ocean mixed layer heat budget in Fig. 2 but for the period of 2019-2023, during which there was no inconsistency issue (i.e., GLORYS12V1 is forced by ERA5). Overall, the results (Supplementary Fig. 17) are similar to those for the entire period (Fig. 2), but much noisier due to the shorter period and therefore the smaller sample size being used. Also, we examined the Q_{net}-induced SST tendency over the entire period, as well as separately before 2018 and after 2019. The spatial patterns and seasonal characteristics are overall very similar for those three periods (Supplementary Fig. 18). These tests provide us additional confidence in the robustness of our results, although they do not completely eliminate the inherent uncertainty in interpreting the residual term. Therefore, ERA5 is used consistently in this study to derive AR-related surface heat fluxes and atmospheric variables across the full analysis period, ensuring a uniform atmospheric reference framework."

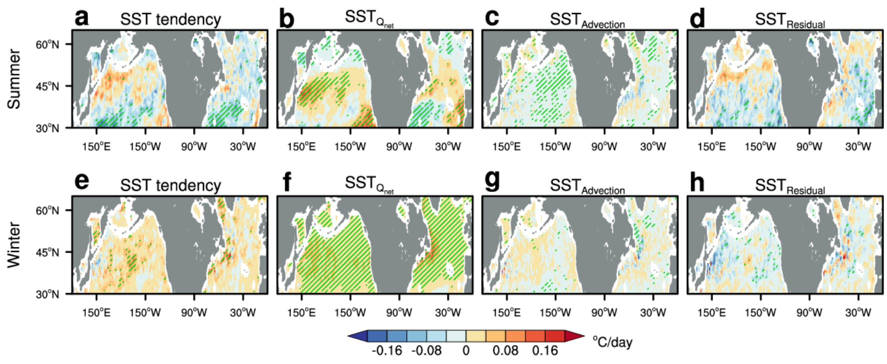

Fig. R4. (a–d) Composite SST tendency (°C/day) and the contributions from net surface heat flux (Q_{net}), horizontal oceanic advection, and the residual term during summer AR days over the period 2019–2023. (e–h) Same as (a–d), but for winter AR days. Hatched regions indicate areas where composite anomalies are statistically significant at the 90% confidence level.

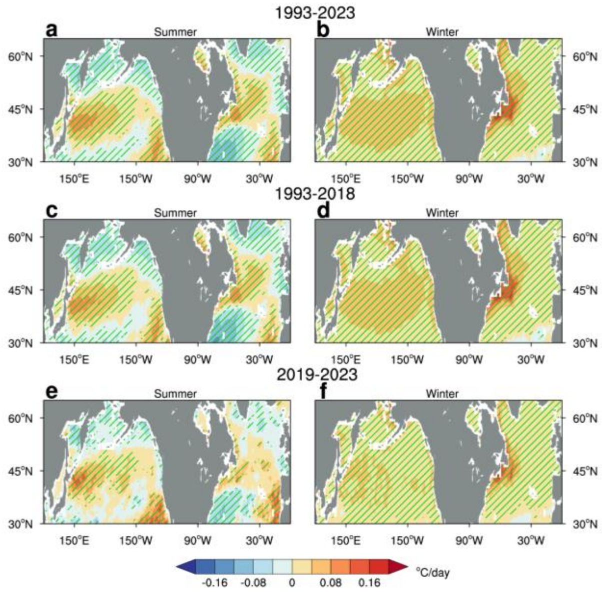

Fig. R5. (a–b) Composite SST tendency (°C/day) contributed by Q_{net} anomalies during summer and winter AR days over the period 1993-2023. (c–d) Same as (a–b), but for the period 1993-2018. (e-f) Same as (a–b), but for the period 2019-2023. Hatched regions indicate areas where composite anomalies are statistically significant at the 90% confidence level.

#4. I am not satisfied with the response to my major comment #2, about the lack of broader synoptic context relevant to summertime heat extremes (e.g. blocking). The added sentence in the discussion section only gestures at the idea, rather than discussing where ARs fall in the broader synoptic context of events that are associated with heatwaves. More discussion of this point is still needed to place the results in a broader context.

Response: We thank the reviewer for this suggestion. In the revised manuscript, we have substantially expanded the discussion (L260-269) as pasted below.

"ARs are closely linked to synoptic atmospheric variability in the extra-tropics, including extratropical cyclones, storm tracks, atmospheric blocking systems, jet streams, etc[46–48]. Our findings highlight AR variability as an important but previously overlooked atmospheric pathway that connects MHW events to these large-scale atmospheric circulation variability in the extratropical oceans. For example, atmospheric blocking, defined as a quasi-stationary high-pressure system, exerts a strong influence on MHWs through reduced wind speed and increased shortwave radiation[40,49]. At the same time, these persistent anticyclonic anomalies can alter large-scale flow and moisture distribution, therefore modulating the propagation and intensity of ARs[48,50,51]. Whether ARs can feed back onto the synoptic circulation variability through moisture convergence and the interactions with MHWs is not fully understood."

Zhang, Z., Ralph, F. M. & Zheng, M. The Relationship Between Extratropical Cyclone Strength and Atmospheric River Intensity and Position. Geophysical Research Letters 46, 1814–1823 (2019).

Payne, A. E. et al. Responses and impacts of atmospheric rivers to climate change. Nat Rev Earth Environ 1, 143–157 (2020).

Benedict, J. J., Clement, A. C. & Medeiros, B. Atmospheric Blocking and Other Large-Scale Precursor Patterns of Landfalling Atmospheric Rivers in the North Pacific: A CESM2 Study. Journal of Geophysical Research: Atmospheres 124, 11330–11353 (2019).

*Amaya, D. J., Miller, A. J., Xie, S.-P. & Kosaka, Y. Physical drivers of the summer 2019 North Pacific marine heatwave. Nat Commun **11**, 1903 (2020).*

Niu, X., Chen, Y. & Le, C. Northeast Pacific marine heatwaves associated with high-latitude atmospheric blocking. *Environ. Res. Lett.* **19**, 014025 (2023).

*Wang, L. et al. Gaps and ways forward in atmospheric blocking and extreme weather research. Nat Commun **17**, 2873 (2026).*

*Bozkurt, D., Marín, J. C. & Barrett, B. S. Temperature and moisture transport during atmospheric blocking patterns around the Antarctic Peninsula. Weather and Climate Extremes **38**, 100506 (2022).*

#5. Several new figures were produced in the R2R but not added to the supplementary data; these would be helpful additions. For example, the results using other AR algorithms should be referenced briefly in the text and added to the supplement, as other readers will likely have similar questions about algorithm-dependence. Other figures explicitly answering reviewer concerns should also be briefly referenced and added to the supplement, including some of the mixed layer budget figures produced in response to Reviewer 1.

Response: We thank the reviewer for this helpful suggestion. In the revised manuscript, we have now incorporated the key additional analyses and figures generated during both the current and the previous revisions.

Line comments.

L26. have emerged

Response: Corrected (L26).

L30. "Seasonal acclimation to ambient temperatures…" I am not sure exactly what this sentence is saying – is it referring to thermal tolerance for all biota? Please clarify.

Response: We thank the reviewer for this helpful comment. Our intention was to convey that many marine species tend to acclimate to the prevailing seasonal temperature conditions, which can reduce their tolerance to temperature anomalies relative to that seasonal baseline. We did not intend to imply that this applies to all biota, but rather that many marine species such as seaweeds, fish, and corals exhibit seasonal dependent physiological responses that make them sensitive to anomalous warming relative to their seasonal environment (seeweed: Lüning, 1984; Atkinson et al. 2020; fish: Bulger and Tremaine, 1985; corals: Berkelmans and Willis, 1999).

To clarify this point, we have revised the sentence in the manuscript as follows (L30-33): “Many marine species acclimate to prevailing seasonal temperatures, which can reduce their tolerance to anomalous warming relative to the seasonal baseline, and even moderate temperature anomalies may thus have important biological consequences.” The related references have also been cited (Lüning 1984; Berkelmans and Willis 1999; Bulger and Tremaine 1985; Atkinson et al. 2020).

Atkinson, J., N. G. King, S. B. Wilmes, and P. J. Moore, 2020: Summer and Winter Marine Heatwaves Favor an Invasive Over Native Seaweeds. Journal of Phycology, 56, 1591–1600, https://doi.org/10.1111/jpy.13051.

Berkelmans, R., and B. L. Willis, 1999: Seasonal and local spatial patterns in the upper thermal limits of corals on the inshore Central Great Barrier Reef. Coral Reefs, 18, 219–228, https://doi.org/10.1007/s003380050186.

Bulger, A. J., and S. C. Tremaine, 1985: Magnitude of Seasonal Effects on Heat Tolerance in Fundulus heteroclitus. Physiological Zoology, 58, 197–204, https://doi.org/10.1086/physzool.58.2.30158567.

Lüning, K., 1984: Temperature tolerance and biogeography of seaweeds: The marine algal flora of Helgoland (North Sea) as an example. Helgolander Meeresunters, 38, 305–317, https://doi.org/10.1007/BF01997486.

L41. Weather systems such as?

Response: We have revised the sentence as (L42): “Besides these low-frequency climate modes, short-lived but intense weather systems, such as tropical cyclones and atmospheric blocking, have also been shown to potentially contribute to the development and persistence of MHWs”. The related reference has been cited (Chen et al. 2014; Neal et al. 2022; Zhou et al. 2025; Rodrigues et al. 2019).

Chen, K., G. G. Gawarkiewicz, S. J. Lentz, and J. M. Bane, 2014: Diagnosing the warming of the Northeastern U.S. Coastal Ocean in 2012: A linkage between the atmospheric jet stream variability and ocean response. Journal of Geophysical Research: Oceans, 119, 218–227, https://doi.org/10.1002/2013JC009393.

Neal, E., C. S. Y. Huang, and N. Nakamura, 2022: The 2021 Pacific Northwest Heat Wave and Associated Blocking: Meteorology and the Role of an Upstream Cyclone as a Diabatic Source of Wave Activity. Geophysical Research Letters, 49, e2021GL097699, https://doi.org/10.1029/2021GL097699.

Rodrigues, R. R., A. S. Taschetto, A. Sen Gupta, and G. R. Foltz, 2019: Common cause for severe droughts in South America and marine heatwaves in the South Atlantic. Nat. Geosci., 12, 620–626, https://doi.org/10.1038/s41561-019-0393-8.

Zhou, X., and Coauthors, 2025: Compound marine heatwaves and tropical cyclones delay the onset of the Bay of Bengal summer monsoon. npj Clim Atmos Sci, 8, 162, https://doi.org/10.1038/s41612-025-01061-5.

L72. The ‘horseshoe’ shape seems less pronounced in the North Atlantic - it is largely negative in lower/western latitudes, and positive in higher/eastern latitudes, with a

much weaker expression in the subpolar regions. This is also seen in the results for other algorithms presented in the R2R.

Response: To address the concern, we have revised the manuscript accordingly to clarify this regional contrast (L76-77): "Over the North Atlantic, similar large-scale structures are found for both seasons but overall weaker. Our analysis and discussion below will consider both basins."

L73. 'basinwide' – see major comment #2.

Response: Please see our response to Comment #2.

L85. Here and elsewhere, remove "firstly," it does not add anything.

Response: Removed.

L89. Fewer, not less, ARs

Response: Corrected.

L93. ~~in fact~~ have already been established

Response: Deleted.

L142. Clarify the sign of the contribution from horizontal advection; perhaps here could add discussion of Ekman transport and relation to budget results in Hsu et al. (2024), as discussed in responses to Reviewer 1. See major comment #1.

Response: We thank the reviewer for this helpful suggestion. As shown in our response to Comment #1, we have clarified the sign of the horizontal advection contribution and explicitly discussed the role of Ekman transport in shaping this term. We also include a direct comparison with the results reported in Hsu et al. (2024).

L171-177. This is all consistent with the same findings in Scholz & Lora (2024), see major comment #1.

Response: In the revised manuscript, we have explicitly acknowledged this connection in the relevant section by adding the following statement (L193-195): "These characteristics are generally consistent with the annual-mean, AR-related surface heat flux anomalies shown by Scholz and Lora (2024), and below we further discuss the distinct features for winter and summer, separately."

L243. Fix punctuation in this sentence, including removing the unnecessary m dash.

Response: Corrected.

L245. Reorder some sentences about the poleward shift/lack thereof here, otherwise it appears to be self-contradicting.

Response: Modified (L270-273).

L250. Again, another unnecessary m dash makes this sentence difficult to parse.

Response: Corrected.

This iteration of the paper is improved in clarity, and I appreciate the additional analyses of the 'horseshoe' pattern seen in summertime, which explains a feature that was not well explained in the original manuscript. However, there are a few remaining issues that the authors did not address adequately. Additionally, the methodological clarifications and statistical significance added to plots have raised some new questions.

Major comments.

#1. The authors did not provide a response for the following line comment, which still stands. To reiterate, the surface flux results presented in Figure 2 (and supplemental figure 6) are extremely similar to those presented in Scholz & Lora (2024), but the authors do not mention this connection in the relevant portion of the text. The discussion of the sign of the radiative and turbulent heat fluxes is likewise very similar to results from this previous study. Comparison of the budget analyses to results in Hsu et al. (2024) are also not made directly in the text, though some discussion was provided in a response to reviewer #1, who had a similar comment. Not including this previous literature as context risks overstating the originality of results, and does not allow for important differentiation between results here with results from previous studies.

Previous comment: L148. These surface flux results are generally consistent with those shown globally in Scholz & Lora (2024), and the budget results have similarities to those shown in Hsu et al. (2024) – comparisons to previous literature would enhance the arguments made here.

#2. Statistical significance. If the purple stippling on Fig 1 (a) and (b) is the significant regions, it seems a stretch to say that the wintertime pattern is 'basinwide,' especially in the North Atlantic. The significance is stronger in the zonal mean (c, d), but on a point-by-point basis (a, b) it is very sparse in coverage and limited to near the coastlines. The data in this plot is also quite noisy – why is this? Additionally, the purple color makes it very difficult to see the stippling on the map.

#3. With the new information about the mixed layer budget requested by Reviewer 1, I now have some questions about the interpretation of the residual term. In the original manuscript, I was under the impression that the surface heat flux terms and the advective terms came from the same source. However, since they are from difference sources, it seems tricky to interpret the residual between the two, as it is possible that part of it may be due to differences in data sources. The authors interpret the residual as an 'oceanic damping' of the atmospheric response, because it is largely opposite in sign to the Qnet term. However, this interpretation relies on the consistency of the two data sources. This should be acknowledged.

#4. I am not satisfied with the response to my major comment #2, about the lack of broader synoptic context relevant to summertime heat extremes (e.g. blocking). The added sentence in the discussion section only gestures at the idea, rather than discussing where ARs fall in the broader synoptic context of events that are associated with heatwaves. More discussion of this point is still needed to place the results in a broader context.

#5. Several new figures were produced in the R2R but not added to the supplementary data; these would be helpful additions. For example, the results using other AR algorithms should be referenced briefly in the text and added to the supplement, as other readers will likely have

similar questions about algorithm-dependence. Other figures explicitly answering reviewer concerns should also be briefly referenced and added to the supplement, including some of the mixed layer budget figures produced in response to Reviewer 1.

Line comments.
L26. **have** emerged

L30. "Seasonal acclimation to ambient temperatures…" I am not sure exactly what this sentence is saying – is it referring to thermal tolerance for all biota? Please clarify.

L41. Weather systems such as?

L72. The 'horseshoe' shape seems less pronounced in the North Atlantic - it is largely negative in lower/western latitudes, and positive in higher/eastern latitudes, with a much weaker expression in the subpolar regions. This is also seen in the results for other algorithms presented in the R2R.

L73. 'basinwide' – see major comment #2.

L85. Here and elsewhere, remove "firstly," it does not add anything.

L89. Fewer, not less, ARs

L93. ~~in fact~~ have already **been** established

L142. Clarify the sign of the contribution from horizontal advection; perhaps here could add discussion of Ekman transport and relation to budget results in Hsu et al. (2024), as discussed in responses to Reviewer 1. See major comment #1.

L171-177. This is all consistent with the same findings in Scholz & Lora (2024), see major comment #1.

L243. Fix punctuation in this sentence, including removing the unnecessary m dash.

L245. Reorder some sentences about the poleward shift/lack thereof here, otherwise it appears to be self-contradicting.

L250. Again, another unnecessary m dash makes this sentence difficult to parse.
